# Discovery of a reversible ALDH1A3 inhibitor through a consensus docking-based virtual screening study

**DOI:** 10.1007/s10822-025-00622-3

**Published:** 2025-08-31

**Authors:** E. Batlle, R. Pequerul, J. Farrés, L. A. Eriksson, K. Pors, V. Jha

**Affiliations:** 1 Institute of Cancer Therapeutics, School of Pharmacy and Medical Sciences, Faculty of Life Sciences, University of Bradford, Bradford, BD71DP UK; 2 Department of Biochemistry and Molecular Biology, Faculty of Biosciences, Universitat Autònoma de Barcelona, 08193 Bellaterra, Barcelona, Spain; 3 Department of Chemistry and Molecular Biology, University of Gothenburg, 40530 Göteborg, Sweden

**Keywords:** Consensus docking, MD simulations, Virtual screening, Cysteine 313/314, Protein–ligand conformational dynamics

## Abstract

Aldehyde dehydrogenases (ALDHs) belong to a group of enzymes that play a vital role in various biological processes and cellular defence against aldehyde toxicity. The ALDH1A subfamily has largely been associated with elevated expression in cancer tissues, and in particular in cancer stem-like cells ALDH1A1 is a frequently expressed enzyme in stem cells and target for therapeutic intervention, however, other isoforms such as 1A2, 1A3, 2, 3A1 and 7A1 have drawn significant attention in the recent years due to their involvement in various pathophysiological conditions. The current study is aimed at therapeutic intervention of ALDH1A3 by developing new inhibitors with the aid of computer-assisted drug design approach. A mixed ligand- and structure-based virtual screening (VS) study employing 4 million compounds, was performed against the X-ray structure of ALDH1A3, supported by hierarchical and consensus docking employing 12 docking solutions, prediction of ADME properties, and binding free energy calculations, to identify new hit and selective compounds. One of the hit molecules (VS1) emerged as a new, and reversible inhibitor of ALDH1A3 from the biochemical screening and kinetic characterization. Molecular dynamics (MD) simulations further allowed us to distinguish between the protein–ligand dynamics of the VS hits and retinoic acid (RA) bound to ALDH1A3, establishing a correlation with the observed experimental results, further comparing with the reported ALDH1A3 inhibitors. VS1 can be used as a good starting point for structure-based hit optimization and hit-to-lead identification. The outcomes of this work are expected to benefit researchers working on computational and rational design of new ALDH inhibitors against cancer and other pathophysiological disorders.

**Graphical abstract:**

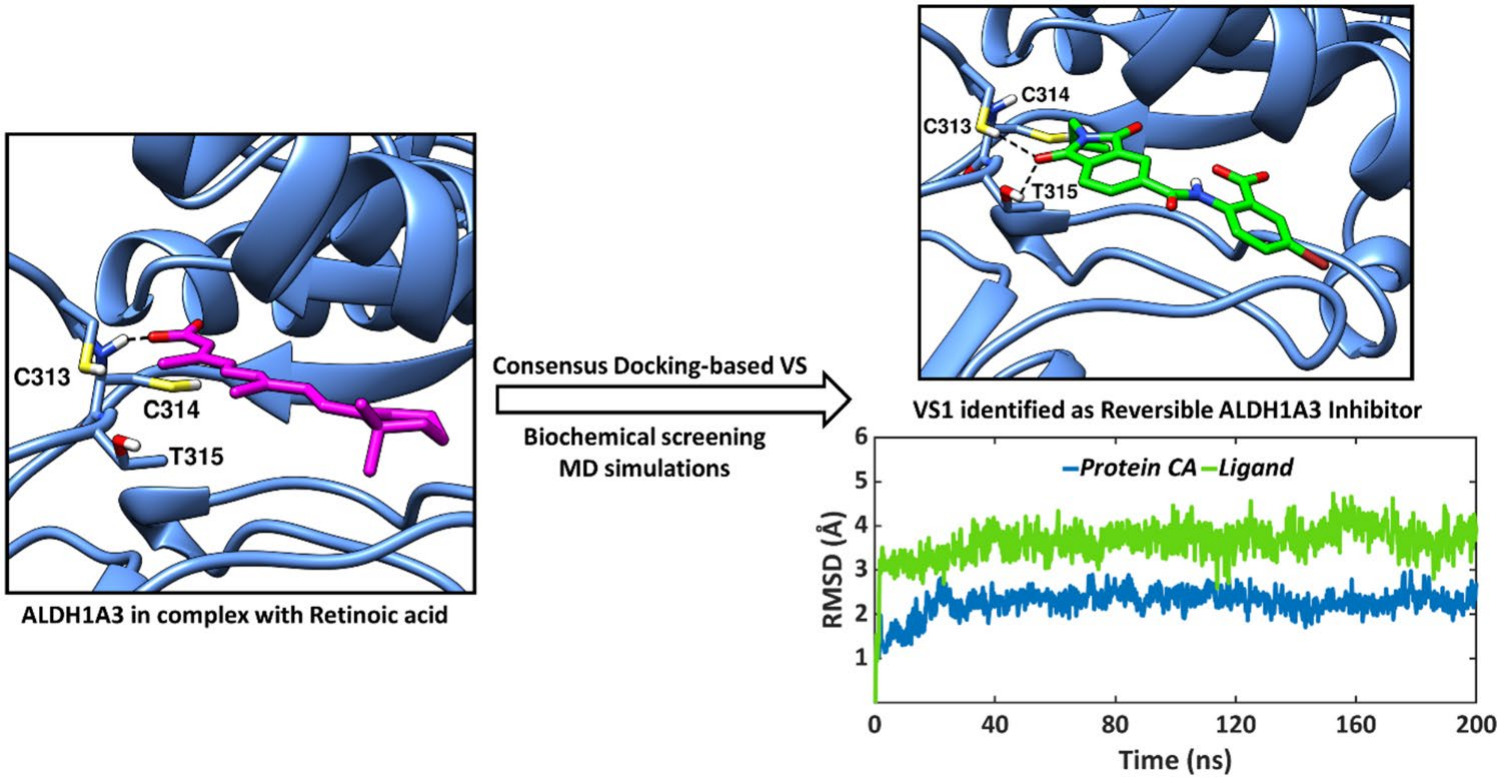

**Supplementary Information:**

The online version contains supplementary material available at 10.1007/s10822-025-00622-3.

## Introduction

Aldehyde oxidases, aldo–keto reductases and aldehyde dehydrogenases (ALDHs) are classes of enzymes responsible for aldehyde metabolism in humans [1]. The human genome is comprised of at least 19 functional genes for ALDHs which catalyse the NAD(P)^+^-dependent oxidation of both endogenous and exogenous aldehydes to their corresponding carboxylic acids or CoA esters [2]. ALDH isoforms can be differentiated by their structure, tissue distribution, subcellular location and preferences for substrates. ALDHs participate in the metabolism of glucose, amino acids and lipids [1] and contribute to various biological functions and cellular defence against aldehyde toxicity [2, 3]. ALDH isoforms are associated with the synthesis of important carboxylic acids such as retinoic acid (RA) and γ-aminobutyric acid (GABA), linked to regulation of cell growth and development [4] and neurotransmission, respectively [5]. Protection of aldehyde-induced cytotoxicity is the principal role of the ALDH family [2]. Over 200 toxic aldehydes including 4-hydroxyhexenal, 4-hydroxynonenal and malondialdehyde are generated due to oxidative stress and lipid peroxidation [6]. These aldehydes may exhibit carbonylation of proteins and have often been linked to neurodegenerative disorders and ageing [7]. Additionally, ALDHs play a regulatory role in initiation and progression of tumours via production of RA from retinal [1]. The formation of DNA adducts with acetaldehyde is an important cause of tumour development, [8] especially in tumours caused by alcoholic beverages [9]. High ALDH expression has been observed in several cancer types such as glioblastoma, [10] epithelial ovarian cancer [11] and melanoma. [12]. Elevated ALDH activity is considered a marker of tumour-initiating cells with stem-like properties, here defined as cancer stem cells (CSC).

Within the ALDH superfamily, the ALDH1A subfamily is important from both a pathological and a metabolic perspective and play a key role in vertebrate development [13]. The ALDH1A subfamily is comprised of the ALDH1A1, 1A2 and 1A3 isoforms. All ALDH1A members are involved in catalysing the second step of RA synthesis by oxidizing *all-trans-* or *9-cis*-retinaldehyde to RA [2]. ALDH1A3 is predominantly responsible for oxidizing *all-trans*-retinaldehyde to RA, with a catalytic efficiency reportedly fivefold higher than the human ALDH1A1 [14]. It is differentially activated during early embryonic head and forebrain development and is highly expressed in the differentiating keratinocytes of human and murine hair shafts [15, 16]. Notably, all distinct isoforms have a specific contribution in different tumour types. ALDH1A1 is a recognized target in human melanoma, [17] whereas ALDH1A2 is a possible tumour suppressor gene in prostate cancer, presumably via the enzyme’s role in retinoid metabolism.

The ALDH1A3 isoform has been reported to be highly expressed in breast cancer, [18] and mesenchymal glioma stem-like cells (GSCs). [19] The histologic subtype of high grade serous ovarian tumours, which is responsible for 70–80% of epithelial ovarian cancers deaths, furthermore illustrated strong elevation of ALDH1A3 expression [11]. In glioblastoma, ALDH1A3 promotes mesenchymal phenotype of GSCs, facilitating aggressive tumour behaviour and therapy evasion [20]. Likewise in breast and ovarian cancers, the overexpression of ALDH1A3 correlates with poor prognosis and increased metastatic potential [21]. Mechanistically, ALDH1A3 leads to drug resistance by detoxifying cytotoxic aldehydes that are generated during oxidative stress or chemotherapy, thereby protecting CSCs from apoptosis and enabling tumour recurrence [20]. Furthermore, the enzymatic activity of ALDH1A3 helps in sustaining redox homeostasis and retinoid signaling, both of which are critical for CSC maintenance [22]. Given its involvement in various pathological conditions including breast cancer, glioblastoma, and epithelial ovarian cancer, ALDH1A3 has emerged as a compelling target for the development of novel therapeutic agents and as a potential prognostic biomarker [23]. Selective inhibition of ALDH1A3 is particularly important, as it may disrupt critical survival mechanisms in cancer cells, enhance tumour sensitivity to standard treatments, and limit the ability of CSCs to drive recurrence [24]. These characteristics position ALDH1A3 as a strong candidate for integration into adjuvant or combination treatment strategies in oncology.

**Fig. 1:**
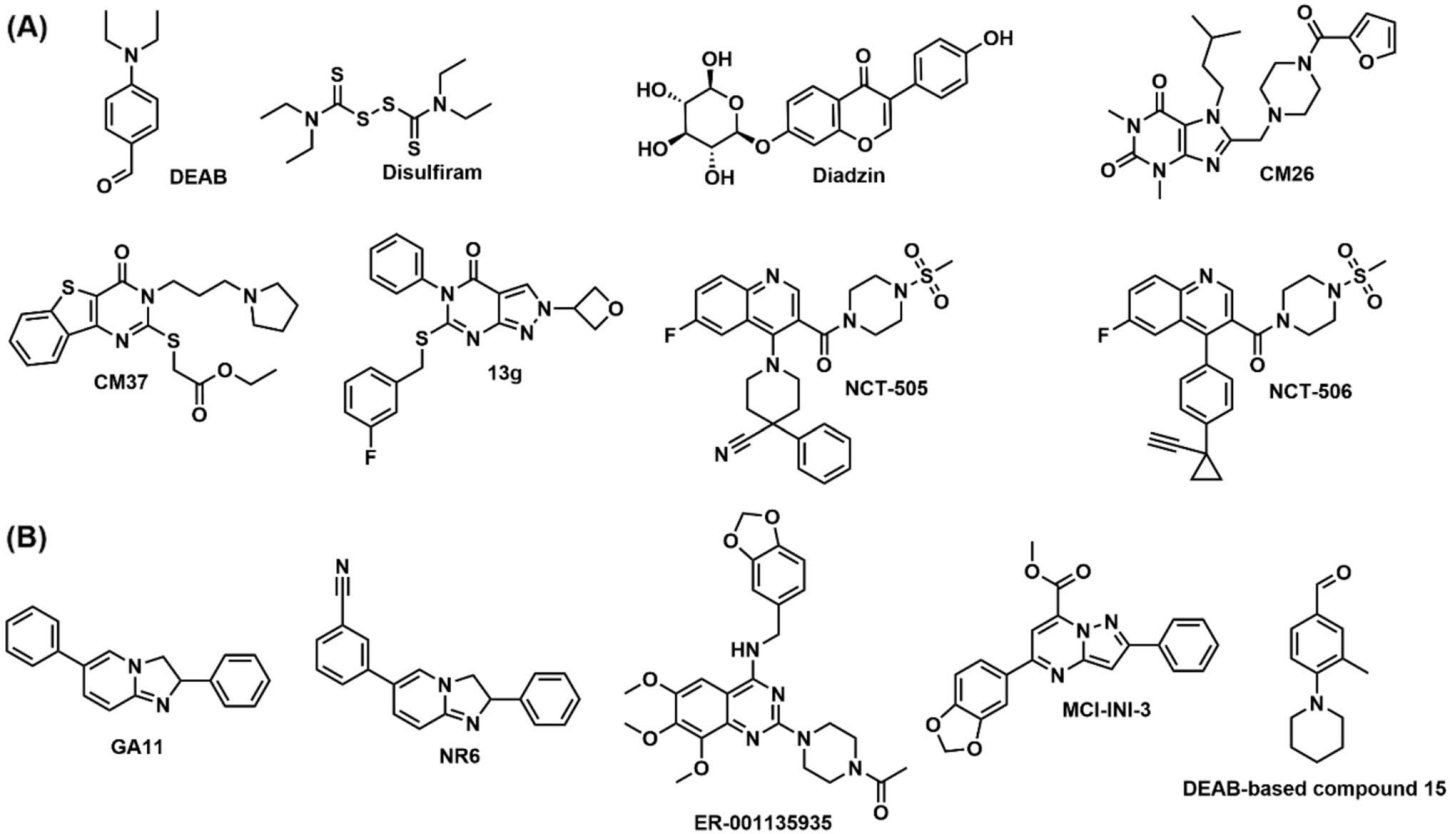
**A** Selection of reported ALDH inhibitors; CM26, CM37, 13 g, NCT-505 and NCT-506 have shown selective ALDH1A1 inhibition. **B** Examples of selective inhibitors of ALDH1A3

Several ALDH inhibitors have been reported in the literature (Fig. 1A). [18, 19] One of the most widely studied ALDH inhibitors is DEAB (N,N-diethylaminobenzaldehyde), which inhibits at least 6 isoforms of ALDH with an IC_50_ < 15 μM [25]. Disulfiram is another nonselective covalent ALDH inhibitor, approved for the treatment of alcoholism, which is rapidly metabolized into several covalent ALDH inhibitors with variable isoform selectivity [18]. A natural product named Diadzin has been reported as a non-selective ALDH inhibitor [26]. Among all ALDH isoforms, ALDH1A1 has been the most explored drug target for anticancer activities. Morgan and co-workers have reported two different chemical classes of non-covalent ALDH1A1 inhibitors by high-throughput screening [27]. The two inhibitors CM026 (dihydropurine-based) and CM037 (benzothieno-pyrimidine-based), representing a specific structural class, exhibits submicromolar inhibition constants and displays ALDH1A1 selectivity over eight other ALDH isoforms [27]. The X-ray structures of ALDH1A1 in complex in CM026 (PDB code: 4WP7) and CM037 (PDB code: 4X4L) has served as excellent starting points for further structure-based drug design studies [27]. Huddle and co-workers developed pyrazole-pyrimidine-based reversible and selective inhibitors of ALDH1A1 from a SAR campaign guided by co-crystallizing one of the HTS hit compounds (CM39) with ALDH1A1 [11]. The inhibitors of this series demonstrated remarkable selectivity against the ALDH1A subfamily over the homologous ALDH2 isoform. The best inhibitor of the series (13 g, PDB code: 6DUM) was found to deplete the CD133^+^ putative cancer stem cell pool, synergized with cisplatin, and was proposed as a potential adjuvant to ovarian cancer chemotherapy [11]. Yang and co-workers have designed a series of quinoline-based orally bioavailable ALDH1A1 inhibitors with excellent enzymatic and cellular ALDH inhibition [28]. In particular, the compounds NCT-505 and NCT-506 inhibited the formation of 3D spheroid cultures of OV-90 cancer cells, and potentiated the cytotoxicity of paclitaxel in SKOV-3-TR, a paclitaxel resistant ovarian cancer cell line [28].

To the best of our knowledge, fewer selective inhibitors of ALDH1A3 have been reported till date, in comparison with ALDH1A1 inhibitors (Fig. 1B). Quattrini and co-workers developed imidazopyridine derivatives from a HTS campaign and kinetic experiments, which demonstrated selectivity for the ALDH1A3 isoform over ALDH1A1 and ALDH1A2 [29]. The best compound of the series (GA11, PDB code: 6S6W) further demonstrated nanomolar to picomolar efficacy against patient-derived glioblastoma stem-like cells [29]. One of the imidazopyridine compounds from the same research group (NR6, PDB code: 7A6Q) was further presented as a highly potent and selective ALDH1A3 inhibitor, and was shown to induce cytotoxic effects and reduction in cell migration and stemness of ALDH1A3-positive cancer cells [30]. The quinazoline-based compound ER-001135935 was identified serendipitously as a selective inhibitor of ALDH1A3 by Kamiyama and co-workers [31]. This study was originally designed to develop inhibitors against type 5 phosphodiesterase (PDE5), however an affinity-based binder identification approach revealed that ER-001135935 possessed selective binding affinity for ALDH1A3 in both cell-based and cell-free assays [31]. Li and workers identified the lead compound MCI-INI-3 as selective competitive inhibitor of human ALDH1A3 (PDB code: 6TGW), showing poor inhibitory activity on the ALDH1A1 isoform [32]. Mass spectrometry-based cellular thermal shift analysis unveiled that ALDH1A3 was the primary binding protein for MCI-INI-3 in GSCs [32]. Ibrahim and co-workers developed 4-diethylaminobenzaldehyde (DEAB)-based compounds that demonstrated some analogues had preferential selective ALDH1A3 inhibitory activity over ALDH1A1 and 3A1 as determined from kinetic assays [33].

Recent progress in the field of computer-aided drug design has significantly boosted the drug discovery process over the years, by identifying the promising drug candidates in early stages of development [34]. Computer-aided drug design tools and techniques such as structure-based and ligand-based virtual screening, molecular docking, pharmacophore modeling and molecular dynamics (MD) simulations are widely used today to discover and refine hit and lead compounds against protein targets of therapeutic interests [35, 36]. These methods allow researchers to explore vast chemical libraries which are otherwise not possible through the conventional drug design routes and gain deeper insights into how the potential drug candidates interact with their protein targets, further facilitating hit-to-lead optimization and lead identification based on the obtained insights [37]. Several studies have highlighted the utility and robustness of the computational strategies in exploiting a variety of pharmacological targets and associated pathways [38]. These computational methods are specifically useful where conventional lab-based screening is too expensive or time taking [39]. Another advantage of the computational strategies include prediction of physicochemical and pharmacokinetic parameters (ADMET) of the proposed hit and lead compounds, that would further help finetune the VS library, eventually prioritizing the most promising drug candidates with optimal ADMET features [40]. Another important aspect of computational modeling techniques involves identifying selective hit and lead compounds against therapeutic protein targets, that further open the doors for the development of targeted-specific therapies, drug delivery and personalized medicines [41]. ALDH1A3 has been targeted in the current study due to its broad-spectrum implication in various types of cancer as mentioned above, indicating its elevated expression as target for anticancer drug development [11, 18, 19]. Only a few number of selective ALDH1A3 inhibitors have been discovered so far, hence the field could benefit from research focused on identifying new chemotypes. Computational modeling thus presents a valuable opportunity to uncover new chemical scaffolds with selective binding properties, offering a strategic edge in the search for novel treatments.

In this context, we have employed computer-assisted drug design, a mixed ligand and structure-based virtual screening (VS) study to identify new, potent and selective inhibitors of ALDH1A3. A hierarchical docking workflow supported by consensus docking and prediction of pharmacokinetic properties was implemented in the VS protocol, which was followed by biochemical screening of promising hit compounds. The experimentally tested compounds were furthermore interrogated by molecular dynamics (MD) simulations in order to establish a correlation between observed ALDH1A3 inhibitory activities and protein – ligand conformational dynamics. In addition, the endogenous ligand retinoic acid (RA), and the two reported inhibitors MF13 [73] and MCI-INI-3 [26] bound to ALDH1A3 were also studied by MD simulations for comparative analysis.

## Materials and methods

### Protein preparation

The X-ray crystallographic structures of ALDH1A3 in complex with RA and NAD (PDB code: 5FHZ) [42], imidazopyridine inhibitor MF (PDB code: 6TRY) [73], pyrazolopyrimidine inhibitor MCI-INI-3 (PDB code: 6TGW), [26] ALDH1A1 with quinoline-based inhibitor CM38 (PDB code: 7UM9), [43] ALDH1A2 with inhibitor WIN 18446 (PDB code: 6ALJ) [44], ALDH3A1 with benzimidazole inhibitor 1DD (PDB code: 4L2O) [45] and ALDH7A1 with DEAB (PDB code: 4X0T). [46] Using the protein preparation wizard [47] as implemented in Maestro, Schrödinger, [48] hydrogen atoms were added and the possible metal binding states were generated. The Prime module [49] of Schrödinger [48] was used to add missing atoms, sidechains and loops to the X-ray structure. Protonation states were assigned, tautomeric states were generated for Asp, Glu, Arg, Lys and His at pH 7.0 ± 2.0, followed by H-bond refinement employing the PROPKA module [50] of Schrödinger [48] at pH 7.0. Water molecules with fewer than two hydrogen bonds to non-waters were removed from the X-ray structure. No structural water molecule that could mediate H-bond interaction with the co-crystallized ligand or the catalytic residues were found within the binding site of ALDH1A3. Geometry refinements of the X-ray complex was carried out using the OPLS4 force field [51] in restrained minimization with the purpose of fixing molecular overlaps and strains. The restrained minimization was terminated when the average root mean square deviation (RMSD) of the protein heavy atoms converged to 0.3 Å.

### Ligand-based screening

ChemBridge, Vitas-M, Enamine and Pharmeks databases comprising around 4 million compounds, were used in the virtual screening. OpenEye SMILE substructure search was employed to filter molecules with at least one carboxylic acid group. A conformational database of 500 conformers per ligand was generated using OMEGA2. [52] Further, employing RA as a query molecule, a ligand-based screening was carried out with the conformational database using ROCS (OpenEye), [53] filtering the compounds with a shape similarity score (shape Tanimoto) not less than 0.65.

### Molecular docking and pose filtering

As per the protocols described by Tuccinardi and co-workers, [54, 55] 12 different docking procedures were employed in this VS study: Autodock 4.2.3, [56] Autodock Vina 1.1, [57] Dock 6.7, [58] Glamdock, [59] Gold 5.1 [60] with its four fitness functions (i.e., ChemScore, GoldScore, ChemPLP and Astex Statistical Potential), Glide 5.0 with standard precision (SP) [61, 62] and extra precision (XP) [63] methods, Plants [64] and rDock. [65] The reliability of each docking program was assessed by performing redocking analysis and calculating the root‐mean‐square deviation (RMSD) between the crystallographic position of the ligand and the ligand’s disposition predicted by docking. The rms_analysis program of the Gold suite was used to calculate the RMSD difference, considering only the heavy atoms of the ligand. All the docking tools were able to reproduce the binding pose of the co-crystallized ligand retinoic acid (RA) bound to ALDH1A3, within an RMSD ≤ 3.0 Å. Similarly, the same redocking analysis was carried out for the Glide SP program, prior to docking virtual screening hit compounds to the other ALDH isoforms (1A1, 1A2, 3A1, 7A1) to evaluate ALDH1A3 selectivity (Figure S15). During the hierarchical docking operation, compounds forming at least one H-bond interaction with C313/C314 were retrieved at the end of this operation.

### Consensus docking study

Consensus docking was performed on the ligands that survived to the 6th docking step and subsequently docked into the ALDH1A3 binding site using 12 different docking methods. In total, 12 top-ranked binding poses were obtained for each of these compounds. The RMSD of each docking pose versus the remaining one was calculated with the aid of the rms_analysis software of the Gold suite and an 11 × 11 matrix reporting the RMSD results was generated. By employing an in-house program, results were clustered based on docking pose similarity. The complete-linkage method was used as a hierarchical clustering algorithm, with an RMSD threshold within each cluster of 2.0 Å. The consensus level of each ligand was defined by the number of docking poses that were clustered together within the 2.0 Å RMSD cut-off and the number of docking methods producing similar binding poses. [54] 54.

### Prediction of physicochemical, pharmacokinetic properties and drug-likeness

In preclinical trials, prediction of pharmacokinetic parameters (ADMET) and estimation of physicochemical parameters play a vital role in verifying the “druggability” of a molecule. The QikProp module [66] in Schrödinger [48] was used to calculate physicochemical and pharmacokinetic properties of the virtual screening hit compounds VS1—VS6. Molecular weight, Lipinski Rule of Five (RO5), octanol/water partition coefficient (logPo/w), aqueous solubility (logS), polar solvent accessible area (PSA), blood/brain partition coefficient (log BB), CNS activity, skin permeability parameter (logKp), blockage of HERG K + channel (logHERG), prediction of binding to serum albumin/plasma protein binding (logKhsa), and percentage human oral absorption (HOA) were computed in the current study.

### Biochemical screening and kinetic characterization

Single-point measurements of enzymatic activity at 10 μM inhibitor concentration (near K_m_ condition for the enzyme) were performed in order to monitor the dehydrogenase activity of ALDH1A3. The enzymatic activity was obtained by following the increase of fluorescence of NADH by sample excitation at 340 nm and emission at 460 nm, respectively. All compounds were dissolved in DMSO and assayed at a final concentration of 1% (v/v) DMSO. The reaction took place in a final volume of 1 mL, in the presence of 10 μM inhibitor, 0.5 mM NAD^+^ and 5 μM NADH as an internal standard, used to obtain absolute reaction rates. As a control, 1% DMSO without added inhibitor was used. The concentration of the enzyme was kept from 50- to 100-fold lower than that of the substrate. Reaction buffer contained 50 mM HEPES (4-(2-hydroxyethyl)−1-piperazineethanesulfonic acid), 50 mM MgCl_2_ and 5 mM DTT (dithiothreitol). In addition to the initial, near K_m_, measurements, the assay was also performed under substrate saturation conditions. This allowed for generation of highly reliable results, as competitive inhibition could be overlooked if a high [S]/K_m_ ratio was used, and similarly for an uncompetitive inhibition if a low [S]/K_m_ ratio was used. By collecting data under the two conditions, the type of inhibition for each compound could be preliminarily predicted. Hexanal was used as the standard substrate for ALDH1A3 and parameters such as the K_m_ and k_cat_ values had already been described [14]. The different substrate concentrations for the assay were 10 μM (near K_m_ condition) and 250 μM (substrate saturation). Stock solutions of hexanal were prepared at a concentration of 2 mM and further diluted to reach the final concentrations required per experiment. The reaction mixture was preincubated for 5 min at room temperature before the substrate was added to initiate the reaction. The results were presented as the percentage of remaining activity, which was calculated as the ratio of the activity in presence of the inhibitor *versus* that of the control with 1% DMSO. In addition, the IC_50_ value was determined for the best inhibitor candidate. Reaction rates were determined at various inhibitor concentrations at a fixed substrate concentration. The IC_50_ value was calculated by nonlinear fitting of the experimental data to a sigmoidal plot using GraFit 5.0 (Erithacus software), as previously described, using 10 μM hexanal as substrate [33].

### MD simulations and clustering

Classical MD simulations were performed using the Desmond program [67] as implemented in the Schrödinger suite [48]. The suggested docking poses of the tested hit compounds from our consensus-docking based VS protocol was used as a starting points in each case. Each protein – ligand complex was solvated in a TIP3P water model. Periodic boundary conditions were applied with a buffer distance of 10 Å around the protein – ligand complex in an orthorhombic simulation box. The total charge of the system was neutralized by adding Na^+^/Cl^−^ counterions. In order to reproduce physiological conditions, a salt concentration of 0.15 M NaCl was added to the protein – ligand simulation box. The default NPT ensemble from the Desmond package was used for minimization and relaxation of each system within the default settings. The OPLS4 force field [51] was applied during all simulations. Each simulation was run for a total of 500 ns with a recording interval of 200 ps. The temperature and pressure of the system were maintained constant at 300 K and 1.01325 bar atmospheric pressure, employing the Nose–Hoover thermostat and Martyna-Tobias-Klein barostat with isotropic coupling [68–70], respectively. Employing the simulation interaction diagram (SID) panel as implemented in Maestro, Schrödinger [48], the RMSD and the protein – ligand interactions were analysed. The “Desmond Trajectory Clustering” module was used to cluster the MD trajectories based on RMSD while setting up a frequency value of 10 (every 10th ns) and generating up to a maximum of 5 clusters. The most populated cluster obtained from the trajectory clustering of each ALDH1A3 – inhibitor complex was used as the representative structure.

### Binding free energy calculation

Molecular mechanics with generalized Born and surface area solvation (MM-GBSA) is a physics-based method, widely used to estimate the binding free energy of a ligand bound to a protein [71]. The Prime module [49] in Schrödinger suite 2022–1 [48] was used to compute MM-GBSA free energy of binding (ΔG bind) for the Glide SP docking poses of the virtual screening hits VS1 – VS3 bound to ALDH1A3 using the following equation:

$$\Delta G\;bind = E_{{Complex}} {-}E_{{Ligand}} {-}E_{{\text{Re} ceptor}} ,$$

where E_Complex_, E_Ligand_, and E_Receptor_ represent the energies calculated in the Prime MM-GBSA module for the optimized complex (complex), optimized free ligand (ligand), and optimized free receptor (receptor), respectively. The OPLS4 force field [51] and VSGB solvation model [72] were employed in the calculations, involving minimization of protein–ligand complexes as the sampling method. The binding free energies (ΔG Bind), the van der Waals energy contribution (ΔG van der Waals) and the Lipophilic energy contribution (ΔG Lipophilic) of each ligand were extracted and are discussed in this work.

## Results and discussion

### Ligand-based screening

**Fig. 2:**
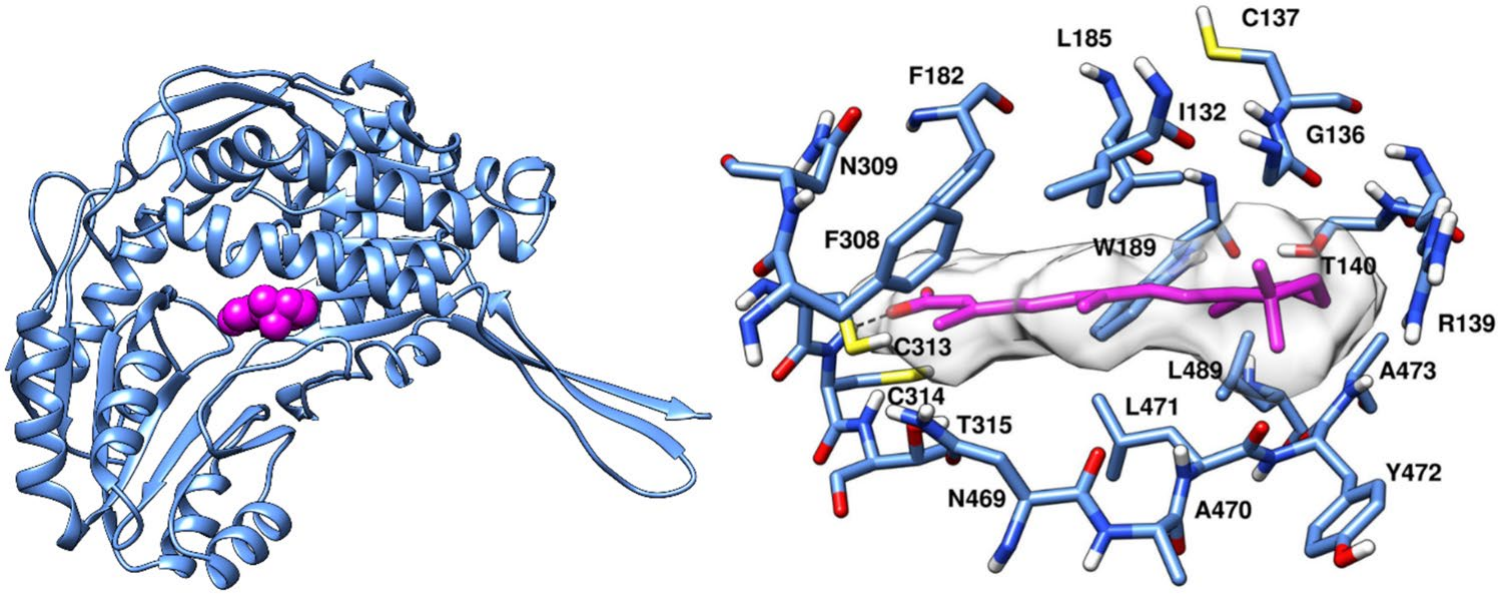
Crystallographic binding mode of RA (magenta) within Chain A of ALDH1A3 monomer (PDB ID: 5FHZ). **A** Ribbon view. **B** Binding site view

ALDH1A3 exists as a tetramer (figure s1), however each monomer is able to convert one molecule of substrate (retinal) to one molecule of product (RA) by reducing one NAD^+^. The X-ray structure of ALDHA3 in complex with RA and NAD^+^ was retrieved from the Protein Data Bank (PDB code: 5FHZ) [12]. Only chain A of ALDH1A3 X-ray structure was used in our VS study while all other chains were deleted (Fig. 2A). RA displayed an extended conformation with its carboxyl group pointing toward the catalytic C314 residue and the isoprenic chain of RA is oriented away from the active site toward the protein surface, so that the entire RA molecule efficiently occupies a tunnel providing substrate access to and product release from, the catalytic cysteine. As depicted in the crystallographic binding mode (Fig. 2B), RA forms a crucial H-bond interaction with C313, while its cyclohexene ring that is projected away from the binding site, establishes van der Waals contacts with residues G136, R139, W189, L471 and A473. These protein–ligand interactions were taken into account during the VS workflow. Initially, a ligand-based strategy was employed to screen a large set of compounds. Since the carboxylic acid of RA H-bonds to C314 (that is hypothesized to contribute to the binding affinity for ALDH1A3), molecules possessing at least one carboxylic acid group were targeted during the pre-screening operation.

Around 4 million compounds from four different chemical databases (ChemBridge, Vitas-M, Enamine and Pharmeks) were pre-screened using OpenEye SMILE substructure search, in order to filter molecules with at least one carboxylic acid. The selection of this VS library was based on a criterion that instead of using just one database, we replied on combining multiple databases that are well-established repositories of drug-like and lead-like small molecules and comply with important medicinal chemistry aspects such as the Lipinski Rule of Five, synthetic accessibility and structural diversity, with reference to our previously successful VS campaigns aimed at different protein targets. [73, 74] Each of these VS libraries is widely used in early-stage drug discovery programs and can thus be highly useful in identifying new chemotypes for the underexplored protein targets such as ALDH1A3, with desirable binding mode and optimal pharmacokinetic parameters. As many as 132,931 molecules were retrieved from the pre-screening step. A conformational database was created for the pre-screened molecules using OMEGA2 [52] by generating 500 conformers per ligand. A ligand-based screening was performed with the prepared database using ROCS (OpenEye), [53] employing RA as the query molecule. Consequently, 39,597 compounds were selected with a shape similarity score (shape Tanimoto) not less than 0.65.

As mentioned above that the carboxylic acid criterion was primarily used to pre-screen the molecules possessing at least one carboxylic acid, in order to emulate the binding mode of the endogenous ligand RA, forming a key H-bond interaction with the catalytic residue C314 at the ALDH1A3 binding site, through its carboxylate. However, it was found during the docking process (discussed below) that not all selected compounds engaged C313/C314 via the carboxylic acid moiety. In fact, possibly due to the structural diversity of the VS library, alternative functional moieties such as the carbonyl group of the isoindoline-1,3-dione scaffold (VS1 – VS3, discussed below), were found to interact with catalytic residues, compensating for the absence of a carboxylate interaction in some cases. While carboxylates were strategically prioritized, other groups were not excluded later in the docking hierarchy, as long as they contributed to the favourable interactions at the ALDH1A3 binding site, particularly forming H-bonds with C313/C314.

### Hierarchical and consensus docking

A hierarchical docking procedure supported by consensus docking, was applied to the 39 597 screened molecules. This hierarchical approach facilitates significant reduction in computation time by employing faster docking methods first and further filtering molecules through a qualitative post-docking filter prior to applying slower docking procedures. [73] The number of ligands analysed were thus significantly reduced at each docking step, allowing the use of slower docking programs in the VS protocol. Starting from the fastest docking method (i.e., Glide SP), the screened molecules were docked and the top-ranked docking pose from each molecule was subjected to filter analysis, selecting only molecules showing the H-bond interactions with C313/314. Compounds that were not able to meet this criterion were rejected, while the remaining ligands were subjected to docking by the next docking method and analysed again for H-bonds with C313/C314. The criteria for a favourable interaction were based on geometric and chemical parameters commonly accepted for inter-molecular H-bonding i.e., donor–acceptor distances ∼ ≤ 3.5 Å and appropriate angular orientation ∼ > 120°) between the donor hydrogen and acceptor atoms. These interactions were evaluated visually and computationally using Maestro, Schrödinger computational platform [48], with reference to the co-crystallized pose of RA, which H-bonds to C314 of ALDH1A3 through its carboxylate group. If a molecule did not form H-bond to C313/C314 in the current docking method, it was excluded for the next docking method. This procedure was repeated for all the remaining docking programs and at the end of 6th docking procedure, 80 compounds were retained.

It is worth noting that some of the compounds among 80 selected hits form H-bond interactions with the catalytic C313/C314 engaging through other portions of the chemical scaffold such as the carbonyl of the isoindoline-1,3-dione among compound VS1—VS3 (discussed later), instead of their carboxylic acid group, acknowledging the fact that H-bond formation does not necessarily rely on the carboxylic acid, instead other chemical scaffolds and functional groups can also form similar interaction, considering the structural diversity and drug-likeness of the VS library used in the work. In addition, the carboxylic acid present among the new hit compounds orients opposite to C313/C314, thereby establishing additional H-bond contacts that contribute to the improvement in the binding affinity for ALDH1A3. Thus, at this stage, we prioritized compounds forming H-bond interactions with C313/C314, not necessarily via their carboxylic acid unit. The selection criteria was thus broadened to have at least one H-bond contact with C313/C314 via either carboxylic acid or any other scaffold in the hit compounds.

Table 1 represents the details of filtration of compounds after each docking step from the first 6 docking procedures. At this stage, the hierarchical docking and further filtering of compounds were stopped since a significantly low percentage of molecules (0.20%) were achieved, which could be subjected to consensus docking analysis. Therefore, the resultant 80 compounds were further docked by employing the remaining docking programs Autodock 4.2.3. [56], Dock 6.5 [58], Glamdock 1.0 [59], Gold 5.1 (GoldScore) [60], rDock 1.0 [65] and Vina 1.1 [57].

**Table 1:** Number of compounds filtered through hierarchical docking procedure

Step	Docking Procedure	Initial no. of Hits	Final no. of Hits (showing H-bonds with C313/C314)	Residual Percentage
1	Glide SP	39,597	2849	7.19%
2	Gold ASP	2849	559	1.41%
3	Gold XP	559	364	0.91%
4	Plants	364	207	0.52%
5	Gold Chemscore	207	90	0.22%
6	Gold PLP	90	80	0.20%

Using the program developed by Tuccinardi and co-workers, [54, 55] we integrated 12 docking solutions through a consensus docking approach that did not rely on explicit weighting or numerical averaging of docking scores (as discussed in the Materials and Methods section). Instead, the methodology was focused on assessing the structural convergence of binding poses across the different docking programs. For each ligand, the root mean square deviation (RMSD) between all pairwise docking poses was calculated, generating an 11 × 11 RMSD matrix. These poses were then clustered using complete-linkage hierarchical clustering with a 2.0 Å RMSD threshold. The consensus level for each compound was defined by the number of docking poses clustered within this threshold and the number of different docking programs contributing to these similar poses. This pose-based consensus strategy prioritized ligands that demonstrated consistent binding conformations across multiple docking algorithms.

**Fig. 3:**
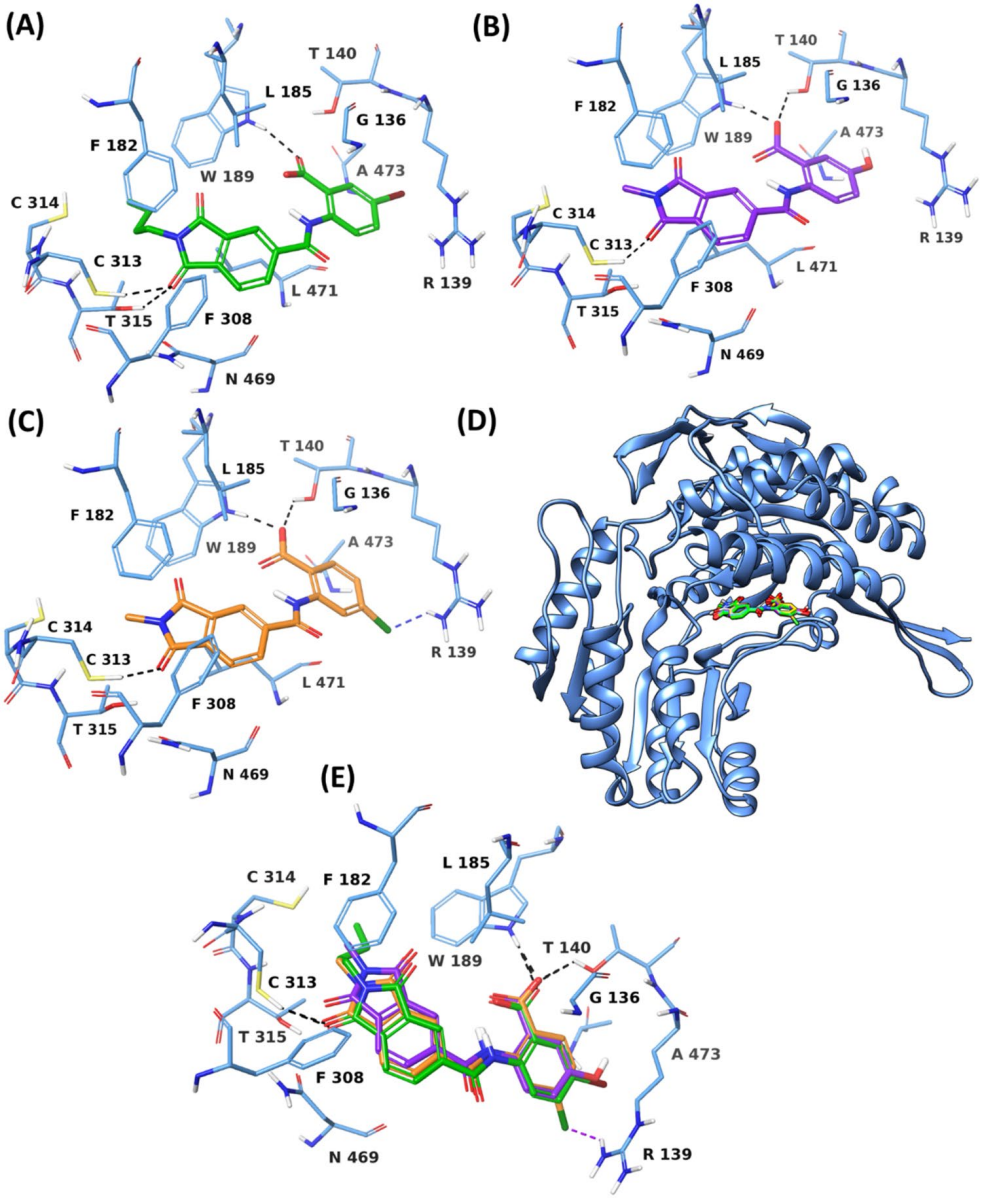
Docking poses of **A** VS1 **B** VS2 **C** VS3, at the ALDH1A3 binding site. Superimposed VS1 -VS3 docking poses in **D** Ribbon view and **E** Binding site view. Protein ribbons/residues are shown in blue, while VS1, VS2 and VS3 are shown in green, purple and orange, respectively

The selected 80 compounds were subjected to a consensus docking platform which involved clustering the 12 different docking poses obtained for each ligand based on their reciprocal RMSD, with a cut-off of 2.0 Å. By applying the consensus docking filter, 34 compounds showing common binding mode from at least 9 out of 12 docking programs were selected for the next steps of the VS workflow, thereby assuming high reliability in their binding modes (Table 2). Despite all the compounds already showing H-bond interactions with C313/C314, the ligands were further analysed and filtered based on forming additional H-bond and lipophilic interactions in the ALDH1A3 binding site. Six out of 34 compounds were prioritized from the visual inspection, on the basis of either establishing van der Waals contacts in the hydrophobic cavity, similar to RA, or maintaining additional H-bond interactions in the enzyme binding site. All 6 compounds were structurally similar with marginal differences, and shared two chemical scaffolds: isoindoline-1,3-dione and benzoic acid (Figure S2). With the structural similarity and as observed from the suggested docking poses (Figs. 3, S3 and S4), the prioritized 6 compounds demonstrated a similar interaction profile at the ALDH1A3 binding site.

**Table 2:** Consensus docking results

Consensus level	No. of compounds	Consensus level	No. of compounds
*12	*2	6	11
*11	*3	5	3
*10	*14	4	3
*9	*15	3	3
8	13	2	0
7	13	1	0

^*^34 compounds from consensus docking level 9 or better were selected

### ADME properties and drug-likeness

The majority of drug candidates often fail during the preclinical stages of the drug development process as a result of poor ADME features such as absorption and extensive first-pass metabolism. Rate of failure of potential drug candidates has reportedly been shown to be reduced by executing an early pharmacokinetic screening. [75] Accurate data on the physicochemical and ADME parameters thus facilitate prioritizing appropriate drug-like molecules as well as providing useful guidelines for dosage form design. To this end, the relevant descriptors of physicochemical and pharmacokinetic properties molecular weight, Lipinski Rule of Five (RO5), logPo/w, logS, PSA, logBB, CNS activity, logKp, logHERG, logKhsa and percentage human oral absorption (HOA) were selected and calculated for the VS hit compounds VS1 – VS6 (table s1). All compounds adhere to the Lipinski Rule of 5, possessing molecular weight <500. The octanol/water partition coefficient (logPo/w) of VS1 – VS6 were between 1.2 and 3.6, indicating that all compounds possessed ideal lipophilic properties. The aqueous solubility (logS) ranged between − 5.5 and − 3.7 mol/L, which is within the recommended values. The polar solvent accessible area (PSA) represents the ability of a compound to interact with the solvent by dipolar or H-bond interaction. The VS1 – VS6 range measurements between 129Å to 164Å of PSA were observed to fall within the acceptable limit. The brain/blood partition coefficient (logBB) of all molecules were in the range from − 2.4 to − 1.4, which is within the tolerable limit. None of the compounds were suggested to have CNS activity (value − 2.0), which is within the tolerable limit. The skin permeability parameter (logKp) denotes the ability of a compound to penetrate through the skin; all molecules span between − 5.6 to − 4.0, which is within the ideal range. We also computed the prediction of blockage of the HERG K+ channel (logHERG), representing cardiac toxicity of drug-like compounds. VS1 – VS6 varied between − 3.9 to − 3.5, which fall within the acceptable values of logHERG. Finally, other ADME features such as plasma protein binding, also known as binding to the human serum albumin (logKhsa) and percentage human oral absorption, that are important for drug-likeness, were taken into account. All molecules showed ideal prediction for binding to the human serum albumin, possessing logKhsa values between − 0.3 to 0.1. The percentage oral absorption of VS1 – VS6 range between 46% and 73% on a 0 −100% scale, suggested mid to high levels of absorption. Taken altogether, the VS hit compounds VS1 – VS6 showed predicted values for all the aforementioned physicochemical and pharmacokinetics parameters within the acceptable thresholds, thus they were further subjected to biochemical screening.

### Binding mode analysis

Based on commercial availability, 3 out of 6 compounds (VS1, VS2 and VS3) were purchased from the respective chemical vendors for the biochemical screening. The individual and superimposed docking poses of VS1—VS3, displaying common binding modes and similar protein—ligand interaction profiles are shown in Fig. 3. The carbonyl of the isoindoline-1,3-dione unit in both VS2 and VS3 form H-bonds to C313 in the ALDH1A3 binding site, whereas that of the VS1 isoindoline-1,3-dione not only H-bonds to C313, but also establishes H-bond interaction with T315. Lipophilic residues F182, F308, and L471 are located in close proximity to the isoindoline-1,3-dione of all three hit compounds, capable of forming van der Waals interactions to improve the binding affinity for ALDH1A3. Moreover, the N-butyl sidechain of the VS1 isoindoline-1,3-dione is in vicinity of the aforementioned residues, thus believed to further improve the binding affinity via lipophilic interactions. The carboxylate of the benzoic acid unit in both VS2 and VS3 H-bonds to T140 and W189, while the carboxylate of the VS1 benzoic acid moiety H-bonds only to W189. Both VS1 and VS2 are characterized by the presence of a *meta*-substituted benzoic acid (VS1 with *m*-bromo, VS2 with *m*-hydroxy), whereas VS2 contains a *p*-chloro substituted benzoic acid. It is also worth noting that the co-crystallized structure of ALDH1A3 used in this study (PDB code: 5FHZ) do not possess any structural or conserved water molecules at the binding site, therefore, the water-mediated H-bonds between the ALDH1A3 and VS hit compounds were not anticipated from the docking analysis.

In order to further validate the consensus docking based strategy, we applied MM-GBSA binding free energy calculations as a rescoring method to the identified hit compounds VS1 – VS3. A halogen interaction was noted between the *p*-chloro group of the VS3 benzoic acid and the R139 sidechain of ALDH1A3, whereas the *meta*-substituted benzoic acid of VS1 and VS2 were found to orient away from R139, showing no interaction. It can be thus hypothesized that the *p*-substituted benzoic acid is preferred to facilitate interactions at the entrance of the ALDH1A3 tunnel, particularly with R139. The aforementioned binding mode and the protein—ligand interactions of VS1—VS3 in the ALDH1A3 binding site further correlate with the docking scores, binding free energy, van der Waals energy and lipophilic energy contribution, as shown in Table 3. VS1 demonstrates the best docking score of −9.47 kcal/mol, relative to − 9.13 kcal/mol and − 8.81 kcal/mol of VS2 and VS3, respectively. Similarly, VS1 shows notably higher binding free energy (ΔG bind) of − 50.50 kcal/mol, in comparison with VS2 (− 41.18 kcal/mol) and VS3 (− 42.08 kcal/mol). The van der Waals (ΔG van der Waals) and the lipophilic energy contribution (ΔG Lipophilic) were likewise found to be significantly higher for VS1 relative to VS2 and VS3. This is in agreement with the presence of N-butyl sidechain in VS1 isoindoline-1,3-dione, which is hypothesized to improve the binding affinity for ALDH1A3. Notably, the lipophilic energy contribution of VS3 is slightly higher than VS2, which can be correlated with the presence of the halogen (*p*-chloro benzoic acid in VS3) [76].

**Table 3:** Glide SP docking scores, MM-GBSA binding free energy results (ΔG bind, ΔG van der Waals and ΔG Lipophilic), in kcal/mol, of VS1 – VS3 in ALDH1A3

Entry	Docking Score	ΔG bind	ΔG van der Waals	ΔG Lipophilic
VS1	− 9.47	− 50.50	− 58.73	− 25.47
VS2	− 9.13	− 41.18	− 53.19	− 18.33
VS3	− 8.81	− 42.08	− 52.42	− 20.71

### Biochemical screening and kinetic characterization

**Fig. 4:**
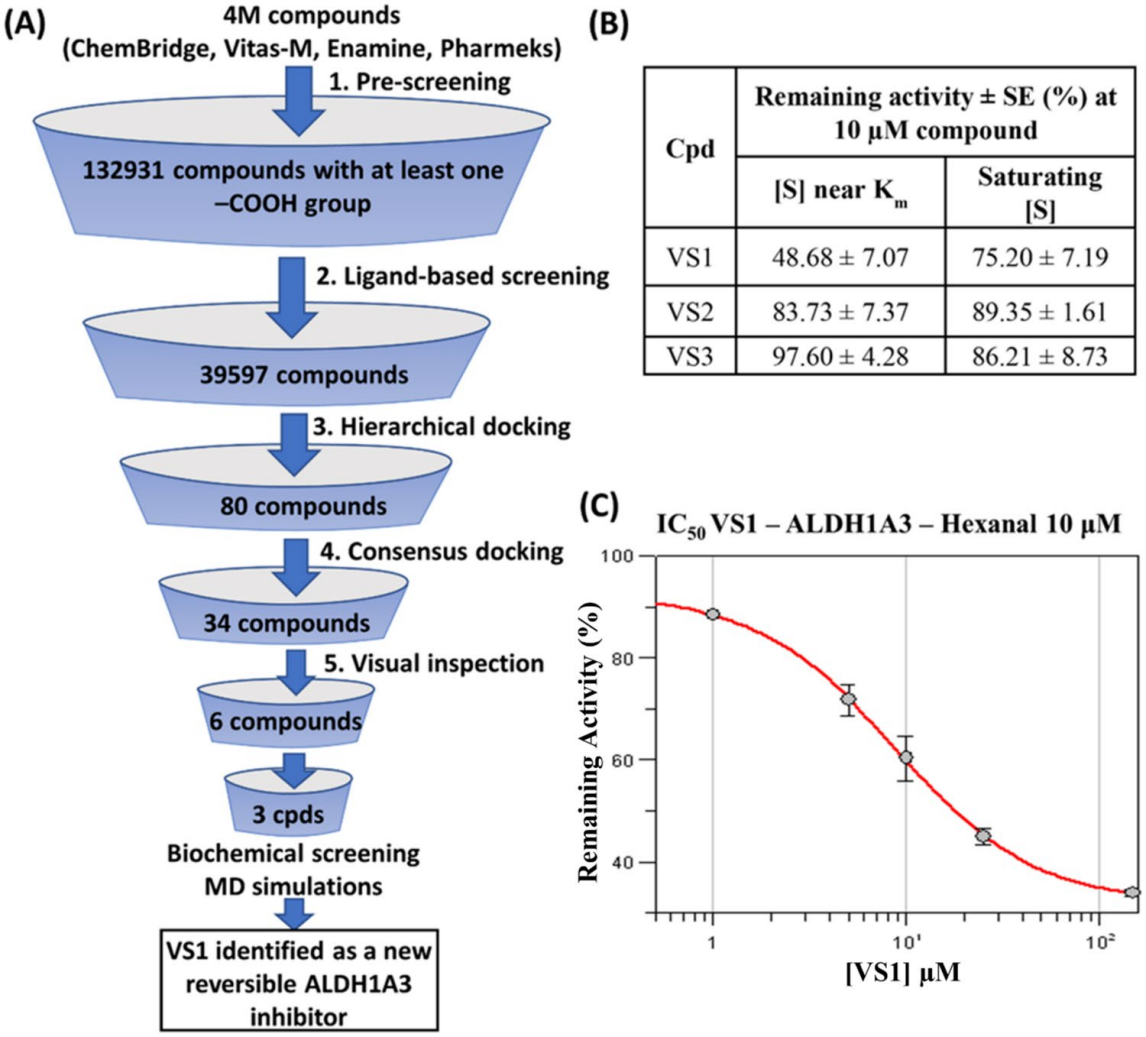
**A** VS workflow. **B** Biochemical screening result. **C** IC_50_ value graph of ALDH1A3 with compound VS1. The percentage of remaining activity is plotted against the logarithm of inhibitor concentration. Data are presented as the mean ± standard deviation from duplicate measurements and the IC_50_ value (8.77 ± 0.45 µM) is shown as the mean ± standard error

The virtual screening hits VS1—VS3 were subjected to ALDH1A3 inhibition screening by monitoring the dehydrogenase activity. The overall virtual screening workflow along with the results of the biochemical screening are illustrated in Fig. 4. Out of the three VS hit compounds, VS1 showed the best values, with a remaining activity of 48.68% and 75.20% at near K_m_ condition and substrate saturating condition, respectively. VS1 was found to be low-micromolar potent, whereas VS2 and VS3 demonstrated poor ALDH1A3 inhibitory activities. Furthermore, VS1 was investigated in a biochemical assay to examine if slow-binding inhibition was occurring, and therefore an irreversible mechanism of ALDH1A3 inhibition could be detected. In this assay, the effect of preincubation on the inhibitory activity of VS1 was assessed. The enzyme was preincubated with the inhibitor for 5 min or 20 min before adding the substrate to initiate the enzyme reaction. Ideally, an irreversible inhibitor should display a higher potency after longer incubation time while a reversible inhibitor should show a constant inhibition potency independent of the incubation time.

VS1 yielded a remaining activity of 75.20% after 5 min preincubation and 68.83% after 20 min preincubation. The results confirmed the reversible nature of VS1 since the inhibitory potency of this compound was only marginally altered at longer incubation time. The binding disposition and corresponding protein–ligand interactions of the low-micromolar potent inhibitor VS1 could be utilized for structure-based hit optimization studies. Following this line, the enzymatic IC_50_ value was calculated for VS1, yielding a result of 8.77 ± 0.45 μM. As per the result, this compound could be a promising candidate for further kinetic characterization. Selective ALDH1A3 inhibitors and their interaction with active-site residues have been recently described (23, 24, 26, 77). They show a reversible binding and a competitive type of inhibition, with comparable IC_50_ values in the low micromolar range (0.46–5.5 µM, Table 4).

**Table 4:** Selective inhibitors of the ALDH1A3 isoform

Inhibitor	PDB code	IC_50_ (µM)	Binding	Type	Reference
GA11	6S6W	4.7 ± 1.7	reversible	competitive	[23]
LQ43	6TE5	3.5 ± 1.2	reversible	competitive	[23]
MF13	6TRY	5.5 ± 1.4	reversible	competitive	[77]
NR6	7A6Q	5.3 ± 1.5	reversible	competitive	[24]
MCI-INI-3	6TGW	0.46 ± 0.06	reversible	competitive	[26]

### MD simulations

#### MD simulations of VS1-VS3 and retinoic acid bound to ALDH1A3

**Fig. 5:**
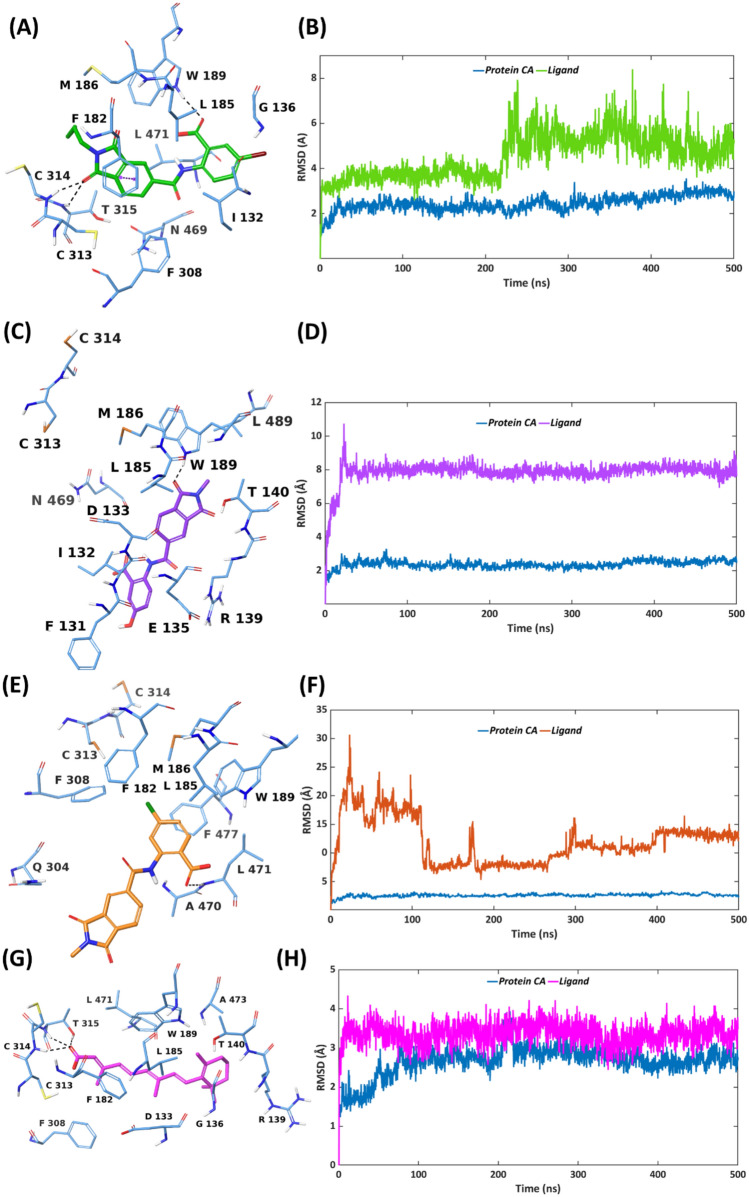
Representative MD structures of **A** ALDH1A3 – VS1 complex (ligand in green, protein residues in blue). **C** ALDH1A3 – VS2 complex (ligand in purple, protein residues in blue). **E** ALDH1A3 – VS3 complex (ligand in orange, protein residues in blue). **G** ALDH1A3 – RA complex (ligand in magenta, protein residues in blue). RMSD analysis of **B** ALDH1A3 – VS1 complex (ligand in green, protein α-carbons in blue). **D** ALDH1A3 – VS2 complex (ligand in purple, protein α-carbons in blue). **F** ALDH1A3 – VS3 complex (ligand in orange, protein α-carbons in blue). **H** ALDH1A3 – RA complex (ligand in magenta, protein α-carbons in blue) during the 500 ns simulation

Despite the similarities in chemical structures, binding modes, and corresponding protein – ligand interactions at the ALDH1A3 binding site, only VS1 was found to have some ALDH1A3 inhibitory potency (IC_50_ value 8.77 µM), while VS2 and VS3 showed poor ALDH1A3 inhibition. With the purpose of further investing the protein – ligand binding at a deeper level, taking into account the flexibility and conformational dynamics of ALDH1A3 when bound to VS1 – VS3, a 500 ns molecular dynamics (MD) simulation was performed for each protein – ligand complex. The co-crystallized ALDH1A3 – RA complex was also subjected to MD simulations in order to compare with the conformational dynamics of the ALDH1A3 – VS1 complex. The Root Mean Square Deviations (RMSD) of the ligand heavy atoms were calculated over the course of the simulation, as a measure of ligand mobility. Likewise, as a measure of protein mobility, the RMSDs of the protein α-carbons were calculated during the simulation. The RMSD represents the deviation of the atoms from their initial crystallographic/suggested docking pose over the course of the simulation. As illustrated in Fig. 5A, B, the ALDH1A3 – VS1 complex demonstrated a significant stability with a minor fluctuation, possessing average RMSDs of 2.33 Å and 3.86 Å for protein and ligand, respectively. The simulation trajectory of ALDH1A3—VS1 can be analysed and elucidated in two halves: (a) 0–216 ns (b) 217–500 ns. During the first half of the simulation, VS1 stays in the binding site of ALDH1A3 in accordance with the suggested docking pose. The H-bond contact between one of the carbonyls of VS1 isoindoline-1,3-dione and the catalytic C314, and T315 of ALDH1A3 exist for 41% and 20% of the simulation time, respectively (Figure S5).

**Fig. 6:**
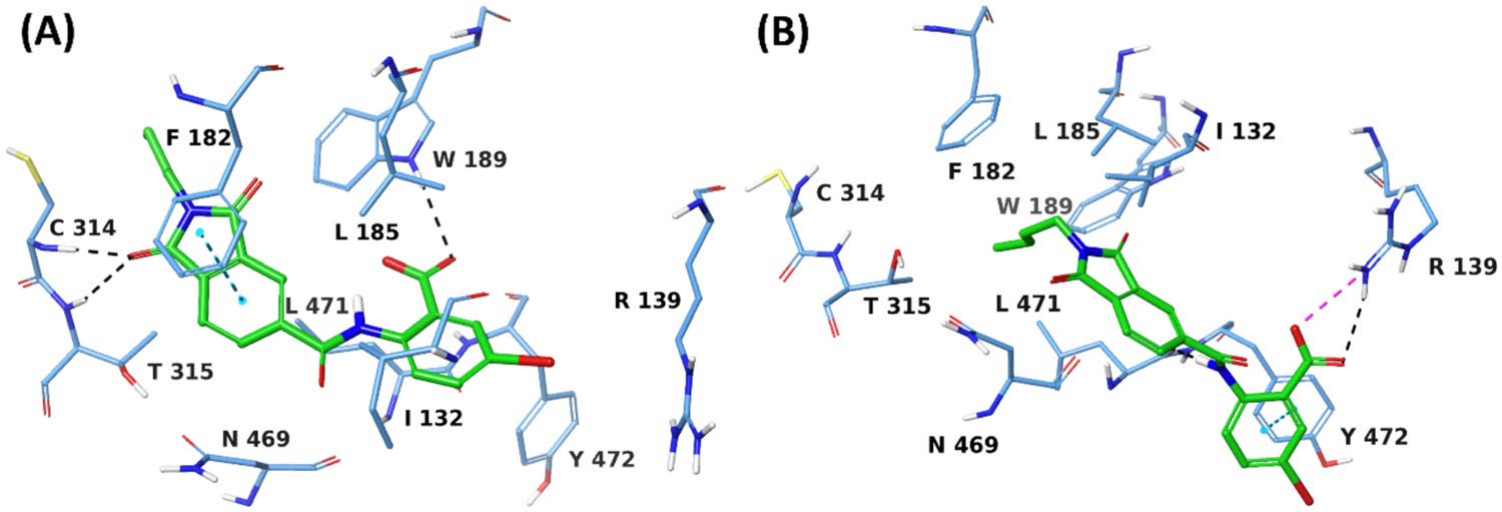
Binding site view from the MD trajectory snapshots of ALDH1A3 -VS1 complex at **A** 58 ns **B** 358 ns of the simulation.

Furthermore, the H-bond contact between the carboxylate of the VS1 benzoic acid and W189 was maintained for 40% of the simulation time. The N-butylated isoindoline core of VS1 contributed to maintaining the van der Waals interactions with lipophilic residues I132, F182, L185 and L471 for 38%, 49%, 22% and 27% of the simulation time, respectively. Figure 6 and Figure S6 illustrate the comparison of the VS1 binding modes during the two halves of the 500 ns simulation. From 0–216 ns (Figs. 6A, S6A), VS1 demonstrated the usual binding mode, interacting with the essential residues of ALDH1A3 binding site, as discussed above. From 217th ns onwards (Figs. 6B, S6B), VS1 is displaced from its original position, yet within the binding site, with its *m*-bromo benzoic acid unit projecting towards the entrance of the ALDH1A3 tunnel.

This conformational change resulted in a couple of interactions: H-bond interactions between the carboxylate of VS1 and R139 maintained for 65% of the simulation time, and π–π stacking between the VS1 phenyl and Y472 for 49% of the simulation trajectory. These additional interactions are thus hypothesized to significantly contribute to the binding affinity for ALDH1A3. Moreover, the N-butyl sidechain linked to VS1 isoindoline is now surrounded by the lipophilic residues I132, F182, L185, W189 and L471 (as discussed above in the overall interaction profile), further improving the binding affinity for the target. We furthermore calculated the Coulomb and van der Waals energy contribution of VS1 bound to ALDH1A3 during the simulation time. The average Coulomb, van der Waals and total (Coulomb + van der Waals) interaction energies of the ALDH1A3 – VS1 complex were found to be −50.32 kcal/mol, − 44.40 kcal/mol and − 94.72 kcal/mol, respectively (Figure S7 – S9). The aforementioned H-bond interactions and the lipophilic contacts between VS1 and ALDH1A3 along with the binding energetics during the simulation trajectory thus confirm a strong binding in agreement with its ALDH1A3 inhibitory potency, as observed from the biochemical screening.

Unlike VS1, both VS2 and VS3 demonstrated significant instability at the ALDH1A3 binding site during the 500 ns MD simulation (Figs. 5C–F). VS2 initially stayed in the ALDH1A3 binding site, maintaining H-bond contact with C313, however, soon after ∼20 ns of the simulation, moved away from the binding site, resulting in loss of H-bond contact with C313 (Figure S10). As a result, one of the carbonyls from VS2 isoindoline-1,3-dione compensated by forming an H-bond with W189 instead of the benzoic acid unit (as observed from the docking pose). This was the only interaction with an essential protein residue (W189), existing for 88% of the simulation time, while the interaction with all other essential ALDH1A3 residues were missing (Figure S11). As evident from the RMSD plot of the ALDH1A3—VS2 complex (Fig. 5D), the protein atoms seemed to be stable, showing an average RMSD of 2.29 Å during the 500 ns simulation, whereas VS2 demonstrated notably higher average RMSD value (7.90 Å), confirming the instability associated with the binding mode of VS2. The hit compound VS3 underwent a complete dissociation from the ALDH1A3 binding site, not maintaining interaction with any of the essential protein residues over the course of the simulation (Figures S12, S13). The protein residues from the ALDH1A3—VS3 complex demonstrated a decent stability, possessing an average RMSD of 2.49 Å, however, the ligand VS3 oppositely exhibits an average RMSD of 16.35 Å, confirming its complete dissociation from the ALDH1A3 binding site. MD analyses of both VS2 and VS3 bound to ALDH1A3 thus correlate with the observed poor experimental inhibitory potency.

To summarize the insights gained from the conformational dynamics of the hit compounds (VS1–VS3) at the ALDH1A3 binding site, it can be concluded that the 500 ns simulation duration was sufficient to draw reliable conclusions. The RMSD plot generated from the 500 ns MD simulation of the ALDH1A3–VS1 complex indicates that sufficient convergence was achieved. Both the protein and ligand RMSDs stabilized early in the trajectory, suggesting a well-equilibrated binding mode, and existence of essential and additional interactions at the ALDH1A3 binding site. Additionally, clustering analysis of the trajectory frames revealed that the majority of conformations sampled during the latter half of the simulation belonged to a dominant cluster, further supporting convergence. The representative MD pose from this cluster is shown in Fig. 5A. On contrary, VS2 and VS3 demonstrated significantly higher and more erratic ligand RMSDs, reaching up to 7.9 Å and 16.4 Å, respectively. These fluctuations indicate unstable or transient binding, which can further be correlated with their lack of ALDH1A3 inhibitory activity, as observed from the biochemical assays. VS2 moved away from the binding site of ALDH1A3 in the early stages of simulation, losing all interactions with the key residues, whereas VS3 was found to completely dissociate from the ALDH1A3 binding site. Therefore, further extending the simulations for these ligands beyond 500 ns was therefore unnecessary, as both VS2 and VS3 clearly failed to maintain stable binding within the simulation timeframe.

Along with the above discussed conformational changes of the ALDH1A3—VS1 complex, the rationale behind the inhibitory activity of VS1 towards ALDH1A3, over that of VS2 and VS3, can be further explained by two distinct structural elements. Firstly, the presence of the N-butyl sidechain at the isoindoline nitrogen of VS1 facilitated additional van der Waals interactions with F182, F308 and L471. Secondly, the presence of the *m*-bromo substituent at the VS1 benzoic acid seems to be favoured over *m*-hydroxy benzoic acid (VS2) and *p*-chloro benzoic acid (VS3). Despite the fact that the *p*-chloro group of the VS3 benzoic acid forms a halogen bond with R139 at the entrance of ALDH1A3 tunnel, it was found in the aforementioned MD analysis that this halogen bond interaction did not really help in stabilizing the ALDH1A3—VS3 complex and can thus be considered as a weak interaction. It can be further hypothesized that the heavier *m*-bromo substitution at the VS1 benzoic acid contributes to stabilizing ALDH1A3 – VS1 complex significantly better than the lighter substituents *m-*hydroxy and *p-*chloro in VS2 and VS3, respectively.

We furthermore carried out 500 ns MD simulations of the ALDH1A3 structure in complex with the endogenous ligand RA in order to compare with the above-discussed conformational dynamics of the ALDH1A3—VS1 complex. Both the protein and RA demonstrated significant stability, possessing average RMSDs of 1.75 Å and 3.45 Å, respectively, during the simulation trajectory, as compared to the average RMSDs of 2.25 Å (protein) and 3.64 Å (VS1) from the ALDH1A3—VS1 complex. Despite being a structurally longer and more flexible molecule, RA stays in the ALDH1A3 binding site entirely Fig. 5G, H. The carboxylate of the RA H-bonds to C314 and T315 for 100% of the simulation time (Figure S14), indicating a great stability in the ALDH1A3 binding site anchoring these residues. As discussed above, the hit compound VS1 demonstrated a similar interaction pattern, forming H-bonds with W189, C314, and T315 and lipophilic interactions with I132, F182, L185 and L471 that were not seen with RA, VS2 or VS3 over the simulation time. Importantly, VS1 demonstrated a crucial conformational change during the course of the simulation, resulting in its displacement from the original binding mode towards the entrance of ALDH1A3 tunnel, establishing a couple of new interactions (H-bond with R139 and π–π stacking with Y472) that are believed to contribute to the binding affinity for ALDH1A3, further correlating with the biochemical results.

#### MD simulations of reported inhibitors MF13 and MCI-INI-3 bound to ALDH1A3

With the purpose of comparing the above discussed MD analysis of the hit molecules VS1-VS3 and the endogenous ligand retinoic acid (RA), bound to ALDH1A3, we furthermore took two external inhibitors into account i.e. MF13 [73] and MCI-INI-3, [26] that can be considered as positive controls in our MD simulation protocol, further validating the conformational dynamics of the identified inhibitor VS1 and its associated ALDH1A3 inhibitory activity. As mentioned above in Table 4, MCI-INI-3 and MF13 have demonstrated reversible and competitive inhibition of ALDH1A3 with IC_50_ values of 5.5 µM and 0.46 µM, respectively [26, 73].

**Fig. 7:**
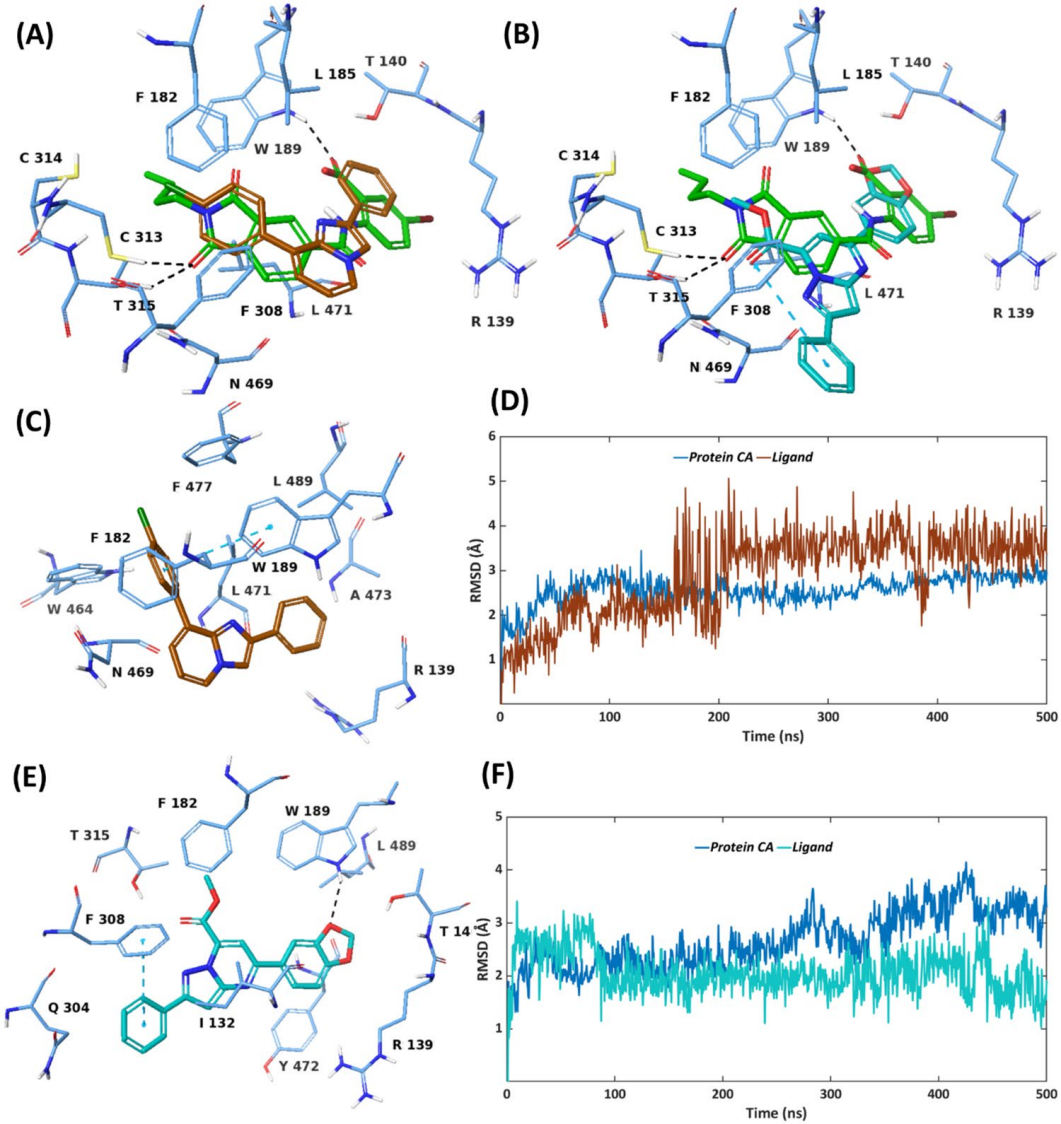
Co-crystallized pose of the reported ALDH1A3 inhibitors **A** MF-13 (PDB code: 6TRY), **B** MCI-INI-3 (PDB code: 6TGW), superposed with the docking pose of the identified inhibitor VS1. MF-13 is colored in brown, MCI-INI-3 in cyan, VS1 in green and protein residues in blue. Representative MD structures of **C** ALDH1A3 – MF-13 complex (ligand in brown, protein residues in blue), **E** ALDH1A3 – MCI-INI-3 complex (ligand in cyan, protein residues in blue) during the 500 ns simulation. RMSD analysis of **D** ALDH1A3 – MF-13 complex (ligand in brown, protein α-carbons in blue), **F** ALDH1A3 – MCI-INI-3 complex (ligand in cyan, protein α-carbons in blue) during the 500 ns simulation

As illustrated in Fig. 7A, the imidazopyridine-based inhibitor MF-13 superposes well with VS1, showing a similar binding mode at the ALDH1A3 binding site. However, unlike VS1, MF-13 is exclusively anchored by the lipophilic contacts as evident from the MD simulations (Fig. 7C). The *p-*chlorophenyl ring of MF-13 forms a strong π–π stacking with W189 of ALDH1A3, existing for ~80% of the simulation time (Figure S16). The same p-chlorophenyl ring is involved in further lipophilic contact with F182 of ALDH1A3, which accounts for ~70% of the simulation trajectory, whereas the central benzimidazole ring system facilitates lipophilic interactions with L471 for 50% of the simulation time. Taken together, despite the lack of H-bond interactions between MF-13 and ALDH1A3, these lipophilic interactions play a crucial role in contributing to the overall binding affinity. MF-13 possesses notable stability at the ALDH1A3 binding site during the course of the 500ns simulation, possessing an average RMSD of 2.89Å, whereas the protein atoms demonstrate an average RMSD of 2.57Å (Fig. 7D). A minor fluctuation between the ~150ns to ~210ns is noted with MF-13, which corresponds to the conformational change that p-chlorophenyl ring of MF-13 undergoes, leading to π–π stacking interactions with both F182 and W189 of the ALDH1A3 binding site. MD snapshots of this conformational change at the 164th and 230th ns of the simulation trajectory are shown in Figure S18. Comparing the crystallographic binding mode and the conformational dynamics of MF-13 with the identified hit inhibitor VS1, it can thus be concluded that VS1 possesses a similar binding mode to that of MF-13 as well as forms essential van der Waals contacts with the lipophilic residues (I132, F182, L185 and L471) at the ALDH1A3 binding site (as discussed in the section “MD simulations of VS1-VS3 and retinoic acid bound to ALDH1A3”.) along with other H-bond interactions (with C313, T315, W189), which overall contribute to its experimental binding affinity of 8.77 µM against ALDH1A3, relative to 5.5 µM of MF-13.

The pyrazolopyrimidine-based inhibitor MCI-INI-3, being a structurally larger molecule, shows an extended binding mode at the ALDH1A3 binding site, relative to VS1 (Fig. 7B). The extended phenyl ring substituent of MCI-INI-3 facilitates a strong π–π stacking with F308, which is maintained throughout the 500 ns simulation (Figure S17). The benzodioxol ring of MCI-INI-3 H-bonds to W189. Moreover, the benzodioxol ring forms a lipophilic interaction with Y472 for ~ 60% of the simulation time, whereas the central pyrazolopyrimidine scaffold of MCI-INI-3 establishes lipophilic contact with I132 for ~ 60% of the simulation trajectory. As observed from the RMSD plot of ALDH1A3 and MCI-INI-3 complex (Fig. 7F), both protein and ligand show significant stability, possessing average RMSD of 2.66 Å and 2.05 Å, respectively. A minor fluctuation with MCI-INI-3 at the beginning of the simulation (~ 10 to 100 ns) corresponds to the rotation of its phenyl moiety which interacts with F308 of ALDH1A3 through lipophilic interactions. The presence of these lipophilic and H-bond interactions between MCI-INI-3 and ALDH1A3 binding site correlates with its remarkable 0.46 µM inhibitory activity, in particular, due to its extended phenyl ring substituent which significantly targets a key nonpolar residue F308 and contributes to the binding affinity for ALDH1A3. Comparing the binding mode and dynamics of MCI-INI-3 with VS1, VS1 H-bonds to W189 of ALDH1A3 through its carboxylic acid, similar to MCI-INI-3. Furthermore, VS1 forms lipophilic contacts with lipophilic residues I132, F182, L185 and L471. In particular, the N-butylated aliphatic group of VS1, attached to the isoindoline-1,3-dione core, facilitates favourable lipophilic interactions with F308, that can further be exploited in our future structure-based hit optimization studies to improve the overall binding affinity.

#### Comparison of the VS1 binding mode with retinoic acid

In order to compare the binding mode of the identified ALDH1A3 inhibitor VS1 with the co-crystallized ligand RA, we superposed the docking pose of VS1 and the co-crystallized pose of RA (PDB code: 5HFZ) bound to the ALDH1A3 (Fig. 8). As previously discussed, the co-crystallized RA H-bonds to C313 through its terminal carboxylic acid group, while the cyclohexene ring of RA occupies space at the entrance of the ALDH1A3 tunnel, establishing van der Waals contacts with residues G136, R139, W189, L471 and A473 of ALDH1A3. Since the RA is the reaction product of retinaldehyde catalysed by ALDH1A3, the newly identified hit molecules, especially bearing a diverse chemical scaffold, are expected to have a different binding mode than that of the endogenous RA. The identified hit compound VS1, being a small molecule, is fully accommodated into the ALDH1A3 binding site; not sitting at the entrance of the ALDH1A3 tunnel similar to RA. Only one H-bond interaction was noted for RA (with C313) deeper within the ALDH1A3 binding site, whereas with VS1, multiple protein – ligand contacts including the H-bond and the lipophilic interactions were observed, which correspond to its 8.77 μM ALDH1A3 inhibitory activity.

**Fig. 8:**
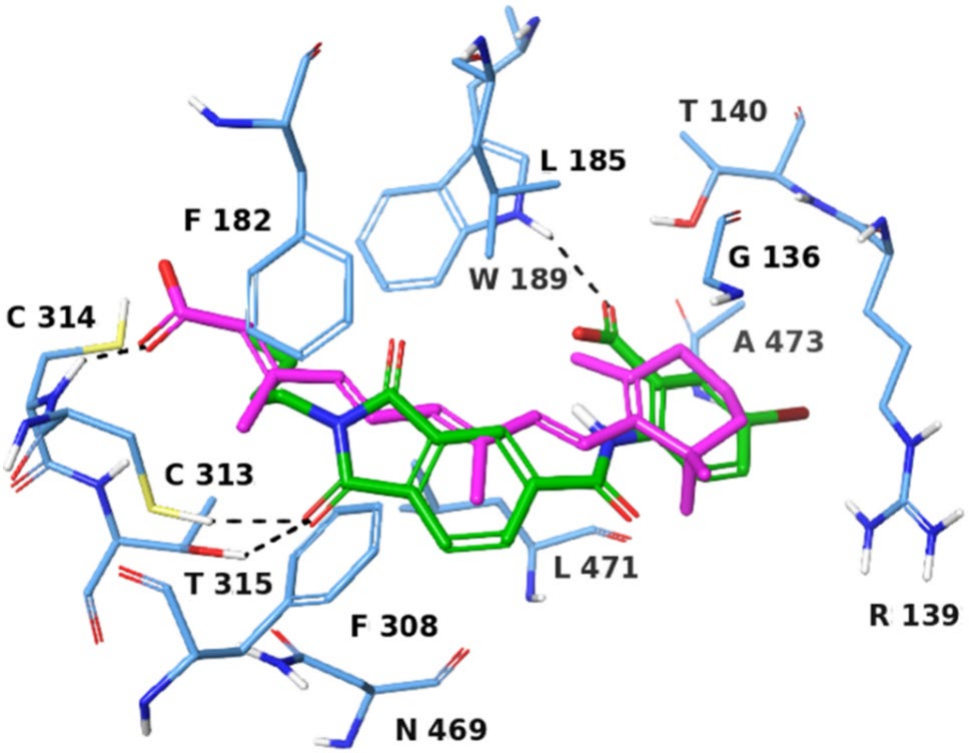
Superposed co-crystallized pose of RA with the docking pose of VS1 at the binding site of ALDH1A3. RA, VS1 and protein residues are shown in magenta, green and light blue, respectively

Taking into account the deeper accommodation into the ALDH1A3 binding site, RA and VS1 can be differentiated by the presence of distinct structural elements: linear carbon chain with the terminal carboxylic acid in RA, leading to one H-bond interaction only, while the presence of bicyclic aromatic ring system (N-butylated isoindoline-1,3-dione) in VS1 facilitating a couple of H-bond interactions (with C313 and T315) and several lipophilic interactions (with F182, F308, and L471) that significantly contributes to the binding affinity for ALDH1A3. In particular, the N-butylated aliphatic group of VS1, appended to the isoindoline-1,3-dione core, introduces additional hydrophobic surface area that facilitates favourable lipophilic interactions with the key nonpolar residues (F182, F308, and L471) encircling the ALDH1A3 binding site. Additionally, the carboxylate of the VS1 benzoic acid H-bonds to the W189 of the ALDH1A3 binding site, which further enhances the binding affinity.

Fascinatingly, these interactions are not only observed in static docking poses but are also maintained for a significant amount of time during the course of 500 ns MD simulation, indicating stable inter-molecular contacts. Further comparing VS1 to the other identified hit molecules (VS2 and VS3), a fundamental structural difference is the absence of this N-butylated sidechain in VS2 and VS3 which leads to reduced hydrophobic complementarity, further correlating with their lower docking scores, poorer binding free energies and eventually weaker inhibitory potency as observed from the biochemical assays. Notably, during the second half of the MD simulation, the N-butyl group of VS1 contributes to a conformational change that allows the ligand to establish a new stabilizing interactions i.e. π–π stacking with Y472 and H-bond with R139, near the entrance of the ALDH1A3 tunnel (Fig. 6). These dynamic rearrangements further enhance the overall binding affinity and explain why VS1 remains stably engaged with the enzyme, whereas VS2 and VS3 exhibit substantial displacement or complete dissociation, and the endogenous ligand RA forms just one H-bond interaction at the ALDH1A3 binding site. The N-butyl sidechain of VS1 can be thus considered as a key pharmacophoric element that promotes both thermodynamic stability and favourable ligand dynamics, making it a valuable feature for future hit optimization.

The binding mode of VS1 is similar to that of selective inhibitors MF13 and MCI-INI-3, which reach deep inside the active-site pocket of ALDH1A3, also interacting with F308 and T315 (77, 26). Like MF13, VS1 also interacts with C314. In contrast, VS1 differs from GA11, LQ43 and NR6, which remain at the entrance of catalytic tunnel (23, 24). Despite this difference, it is predicted that VS1 may be displaced from its original position during the second half of the simulation, also interacting with ALDH1A3-specific Y472, not conserved in other isoforms.

### Assessing the selectivity of VS1 for ALDH1A3 over other ALDH isoforms

Specifically targeting ALDH1A3 can be a strategic approach in cancer drug development because of its implication in cancer progression, metastasis, therapy resistance, and cancer stem cell (CSC) maintenance in specific tumours as such as glioblastomas, breast and epithelial ovarian cancers [18, 19]. Other ALDH isoforms such as 1A1, 1A2, 3A1 and 7A1 share structural resemblance to ALDH1A3, however, they do not necessarily overexpress in the same cancers. Moreover, these isozymes are also involved in normal physiological functions such as non-cancerous stem cells or detoxification pathways [78]. In addition, ALDH1A3 has been reported as a biomarker in the above-mentioned cancer types and can be thus targeted for personalized therapies [19]. It is therefore crucial to evaluate the binding of the identified inhibitor VS1 with respect to other ALDH isoforms, to assess its selectivity and potential off-target effects.

VS1 was docked to the human X-ray crystal structures of ALDH1A1 (PDB code: 7UM9), ALDH1A2 (PDB code: 6ALJ), ALDH3A1 (PDB code: 4L2O) and ALDH7A1 (PDB code: 4X0T). Relative to the remarkable docking score of − 9.47 kcal/mol with respect to ALDH1A3, VS1 demonstrated docking scores of − 6.11, − 3.55 and − 2.45 kcal/mol for ALDH1A1, ALDH3A1 and ALDH7A1, respectively, whereas VS1 did not show any binding to the ALHD1A2 isoform (Table 5). We furthermore compared the docking poses of VS1 with the co-crystallized inhibitors of the respective ALDH isoforms. As illustrated in Fig. 9A and B, VS1 projects outward toward the ALDH1A1 binding site, constituting only a single halogen interaction between the bromine atom of its *m*-bromo benzoic acid and K128. In contrast, the co-crystallized inhibitor CM38, a pyrazolo-pyrimidine derivative, is embedded more deeply within the ALDH1A1 binding site, forming lipophilic interactions with key residues such as Y297, which are known to enhance binding affinity. The peripheral orientation of VS1 and its lack of interaction with key residues explain its lower docking score of − 6.11 kcal/mol and confirm its weak binding affinity for ALDH1A1. Unlike with ALDH1A1, VS1 adopts a binding mode similar to that of the co-crystallized inhibitor 1DD, a benzimidazole derivative, at the ALDH3A1 binding site(Fig. 9C and D). However, VS1 lacks the bicyclic structural features, such as the benzimidazole ring in 1DD, that potentiates π–π stacking with F401. This structural limitation likely contributes to VS1’s weaker docking score of − 3.55 kcal/mol at the ALDH3A1 binding site.

**Table 5:** Glide SP docking scores, in kcal/mol, of VS1 in other ALDH isoforms, relative to ALDH1A3

Entry	ALDH isoforms	Docking Score (kcal/mol)
1	ALDH1A1	− 6.11
2	ALDH1A2	No binding
**3**	**ALDH1A3**	− **9.47**
4	ALDH3A1	− 3.55
5	ALDH7A1	− 2.45

**Fig. 8:**
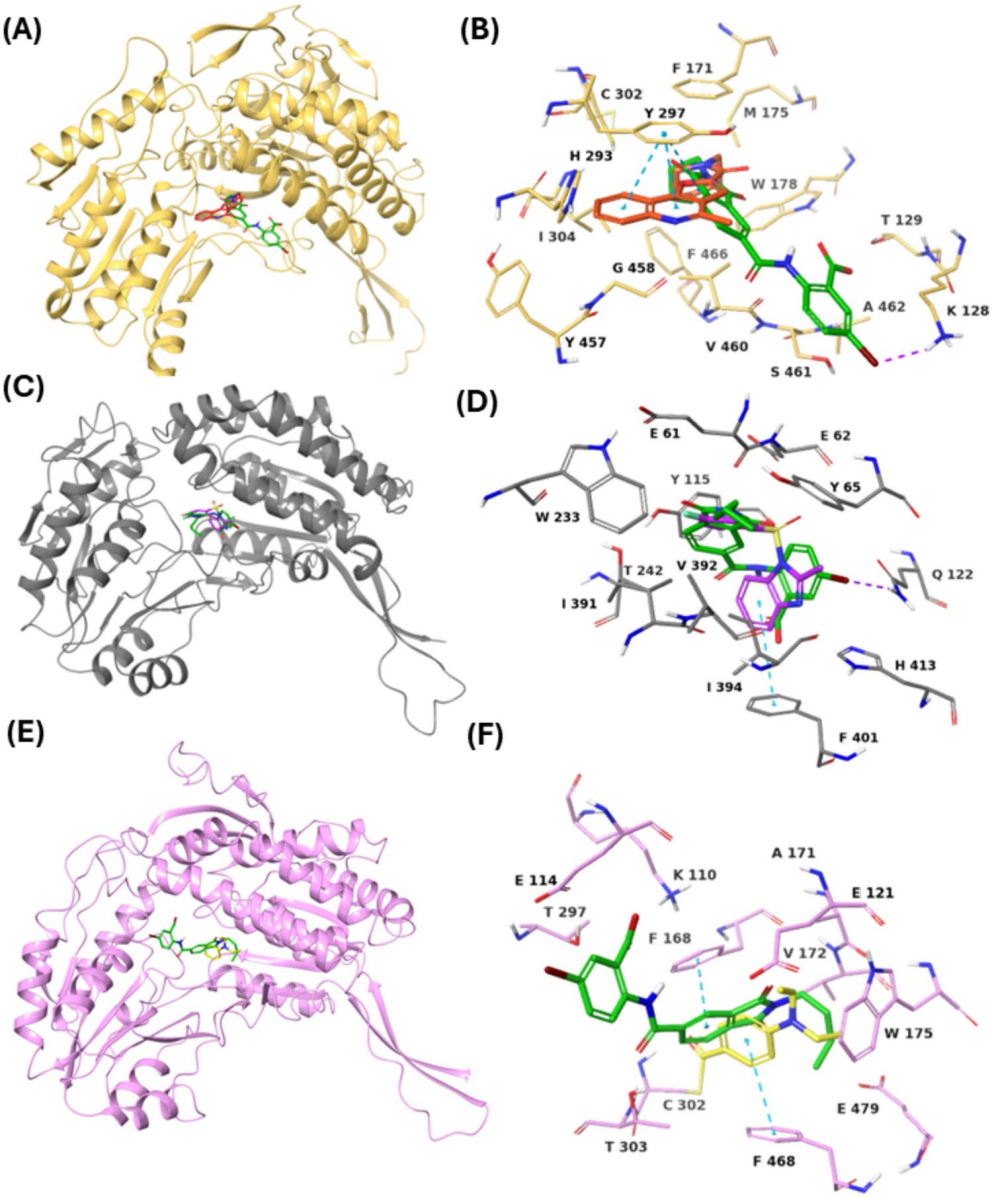
Docking pose of VS1: **A** ribbon view, **B** binding site view, in ALDH1A1, superposed with its co-crystallized inhibitor CM38 (PDB code: 7UM9). ALDH1A1 atoms/ribbons are coloured in yellow, VS1 in green and CM38 in red. Docking pose of VS1: **C** ribbon view, **D** binding site view, in ALDH3A1, superposed with its co-crystallized inhibitor 1DD (PDB code: 4L2O). ALDH3A1 atoms/ribbons are coloured in grey, VS1 in green and 1DD in magenta. Docking pose of VS1 **E** ribbon view, **F** binding site view, in ALDH7A1, superposed with its co-crystallized inhibitor DEAB (PDB code: 4X0T). ALDH7A1 atoms/ribbons are coloured in pink, VS1 in green and DEAB in yellow

Finally, at the ALDH7A1 binding site (Fig. 9E and F), VS1 exhibits a distinct binding mode compared to the co-crystallized inhibitor DEAB (4-diethylaminobenzaldehyde), a well-characterized non-specific covalent ALDH inhibitor. DEAB forms covalent and π–π stacking interactions with residues C302 and F468, respectively, whereas the identified ALDH1A3 inhibitor VS1 is oriented away from the ALDH7A1 binding site, establishing only a single π–π stacking interaction with F168, resulting in a significantly lower docking score of − 2.45 kcal/mol. Taken together, the docking scores and binding mode analyses confirm that VS1 exhibits strong selectivity for ALDH1A3 while demonstrating poor binding to other ALDH isoforms, including ALDH1A1, ALDH1A2, ALDH3A1, and ALDH7A1. This specificity highlights the potential of VS1 as a promising candidate for hit-to-lead optimization aimed at developing isoform-selective cancer therapies and advancing biomarker discovery.

## Conclusion

The ALDH superfamily of enzymes is involved in various biological processes such as synthesis of RA and GABA, contributing to cellular growth and development, and protection from aldehyde toxicity. In relation to cancer and other pathophysiological conditions such as neurodegenerative disorders, the ALDH1A subfamily has shown significant involvement and high expression. The first member of the subfamily i.e. ALDH1A1 is the most studied protein target for the discovery of small molecule inhibitors, however, other members such as 1A2, 1A3, 3A1 and 7A1 have demonstrated great potential to be considered as prominent drug targets. In particular, the ALDH1A3 isoform has gained significant attention due to its overexpression in various types of cancers such as breast, prostate, ovarian cancer, glioblastoma and melanoma. With the aid of computational modeling, we have carried out a mixed ligand- and structure-based virtual screening (VS) study to identify new inhibitors of ALDH1A3. The endogenous ligand RA was used as the starting point in the VS workflow. A combination of four chemical databases (ChemBridge, Vitas-M, Enamine and Pharmeks) comprising 4 million compounds were used as the VS library, in order to offer structural diversity and medicinal chemistry aspects such as the Lipinski Rule of Five, synthetic accessibility and drug-likeness. The VS library was first screened to ensure having at least one carboxylic acid. We initially anticipated that the carboxylic acid would enable H-bond interactions with C313/C314 in the ALDH1A3 binding site, however, it was later found that the carboxylic acid of the selected hit compounds protrude away from C313/C314 establishing additional interaction with the binding site residues, whereas the required H-bond contact with C313/C314 is compensated by other portions of the molecule. The *in-silico* screening protocol contained both hierarchical and consensus docking, employing 12 docking methods, prediction of physicochemical and pharmacokinetic properties and binding free energy calculations, which lead to the identification of three hit compounds (VS1—VS3). All compounds were characterized by an N-substituted isoindoline-1,3-dione and a substituted benzoic acid, displaying desirable binding mode relative to the endogenous ligand RA. VS1—VS3 not only formed fundamental H-bonds with C313/C314 but also demonstrated the existence of other substantial H-bonds (with T140, T315, W189) and lipophilic contacts (with F182, F308, L471) in the ALDHA3 binding site. The hit compounds were subjected to ALDH1A3 biochemical screening and kinetic characterization, revealing that VS1 was the most potent inhibitor with an IC_50_ value of 8.77 μM, while VS2 and VS3 demonstrated poor inhibitory potency. Preincubation experiments furthermore confirmed that VS1 is a reversible inhibitor of ALDH1A3. In addition, the ligand binding mode and the stability of the protein—ligand complexes involving VS1—VS3 and the endogenous ligand RA at the binding site of ALDH1A3 were thoroughly analysed by molecular dynamics (MD) simulations. MD analyses highlighted that VS1 stays in the binding site of ALDH1A3, displaying a desirable binding mode and interacting with essential and additional residues throughout the simulation, which showed key conformational changes. VS2 and VS3, on the other hand, were shown to more distant to the binding site, which was in agreement with the observed experimental results. Apart from the isoindoline-1,3-dione core of VS1—VS3, the *m*-bromo substituent at the VS1 benzoic acid seemed to be largely favoured over *m-*hydroxy and *p*-chloro benzoic acid substituents of VS2 and VS3, respectively. Accordingly, this can be considered as an important structural aspect contributing to the stability of the ALDH1A3—VS1 complex, further correlating with the observed inhibitory potency. The conformational dynamics of VS1 were also compared with the reported ALDH1A3 inhibitors MF-13 and MCI-INI-3, confirming that VS1 possesses a similar/comparable binding mode and shows key protein—ligand interactions at the binding site of ALDH1A3, similar to that of MF-13 and MCI-INI-3. To assess the selectivity of the identified ALDH1A3 inhibitor, VS1 was docked into the binding sites of other homologous ALDH isoforms 1A1, 1A2, 3A1, and 7A1, which are overexpressed in various cancer types as well as in normal human tissues. VS1 exhibited poor or no binding affinity toward these isoforms, confirming its selective interaction with ALDH1A3. This finding is pivotal for future efforts aimed at developing targeted therapies. The outcomes of this study will serve as a promising starting point for optimization studies and further hit-to-lead identification of new ALDH1A3 inhibitors in medicinal chemistry supported programs. Moreover, the extensive computational protocol described herein will benefit researchers working in ligand- and structure-based drug discovery by in silico modeling.

## Supplementary Information

Below is the link to the electronic supplementary material.

Supplementary file1 (PDF 1797 kb)

## Data Availability

The structures of the docked complexes, and the MD trajectory files of VS1 – VS3 and RA with respect to ALDH1A3 and VS1 with respect to ALDH1A1, ALDH3A1 and ALDH7A1 are provided as tarballs (.tar.gz) freely accessible at zenodo.org through doi: 105281/zenodo 11394653.
