# Supplementary material for: Discovery of a reversible ALDH1A3 inhibitor through a consensus docking-based virtual screening study
Source: J Comput Aided Mol Des. 2025 Aug 31;39(1):72. doi: 10.1007/s10822-025-00622-3 (PMC12399719; doi:10.1007/s10822-025-00622-3)
Supplement: Supplementary file 1 — Supplementary file1 (PDF 1797 kb) [file 10822_2025_622_MOESM1_ESM.pdf]

Discovery of a reversible ALDH1A3 inhibitor through a consensus docking-based virtual screening study

E. Battle[1,2], J. Farres[2], Raquel Pequerul[2], L. A. Eriksson[3], K. Pors[1], V. Jha[1*]

[1]Institute of Cancer Therapeutics, School of Pharmacy and Medical Sciences, Faculty of Life Sciences, University of Bradford, Bradford, UK BD71DP

[2]Department of Biochemistry and Molecular Biology, Faculty of Biosciences, Universitat Autònoma de Barcelona, Bellaterra, Barcelona E-08193, Spain

[3]Department of Chemistry and Molecular Biology, University of Gothenburg, 405 30 Göteborg, Sweden

Correspondence:

*Vibhu Jha, Institute of Cancer Therapeutics, School of Pharmacy and Medical Sciences, Faculty of Life Sciences, University of Bradford, Bradford, UK BD71DP
Email: v.jha2@bradford.ac.uk
Phone: +44 (20) 76608328

*Klaus Pors, Institute of Cancer Therapeutics, School of Pharmacy and Medical Sciences, Faculty of Life Sciences, University of Bradford, Bradford, UK BD71DP
Email: kpors1@bradford.ac.uk
Phone: +44 (0)1274 236482

Table of contents

Figure S1. X-ray structure of tetrameric ALDH1A3. P3

Figure S2. 2D chemical structures of virtual screening hits VS1 – VS6. P3

Figure S3. 2D protein – ligand interaction diagram of virtual screening hits VS1 – VS6 and Retinoic acid (RA) in the ALDH1A3 binding site. P4

Figure S4. Docking pose of VS4, VS5 and VS6 in the ALDH1A3 binding site. P5

Table S1. Computed pharmacokinetic and physicochemical properties of the virtual screening hit compounds VS1 – VS6. P6

Figure S5. Protein-ligand interaction histogram from the MD simulations of VS1 in the ALDH1A3 binding site. P6

Figure S6. MD trajectory snapshots of ALDH1A3 -VS1 complex at 58ns and 358ns of the simulation. P7

Figure S7. Van der Waals interaction energy contribution between VS1 – ALDH1A3 during the 200ns MD simulation. P7

Figure S8. Coulomb interaction energy contribution between VS1 – ALDH1A3 during the 200ns MD simulation. P8

Figure S9. Total interaction (Van der Waals + Coulomb) energy contribution between VS1 – ALDH1A3 during the 200ns MD simulation. P8

Figure S10. MD trajectory snapshots of ALDH1A3 -VS2 complex at 0ns and 20ns. P9

Figure S11. Protein-ligand interaction histogram from the MD simulations of VS2 in the ALDH1A3 binding site. P9

Figure S12. MD trajectory snapshots of ALDH1A3 -VS3 complex at 0ns and 20ns of the simulation. P10

Figure S13. Protein-ligand interaction histogram from the MD simulations of VS3 in the ALDH1A3 binding site. P10

Figure S14. Protein-ligand interaction histogram from the MD simulations of Retinoic acid in the ALDH1A3 binding site. P10

Figure S15. Redocking analysis of the co-crystallized complexes of ALDH1A1, ALDH1A3, ALDH3A1 and ALDH7A1. P11

Figure S16. Protein-ligand interaction histogram from the MD simulations of MF13 in the ALDH1A3 binding site. P12

Figure S17. Protein-ligand interaction histogram from the MD simulations of MCI-INI-3 in the ALDH1A3 binding site. P12

Figure S18. MD snapshots of MF-13 conformational changes in ALDH1A3 P13

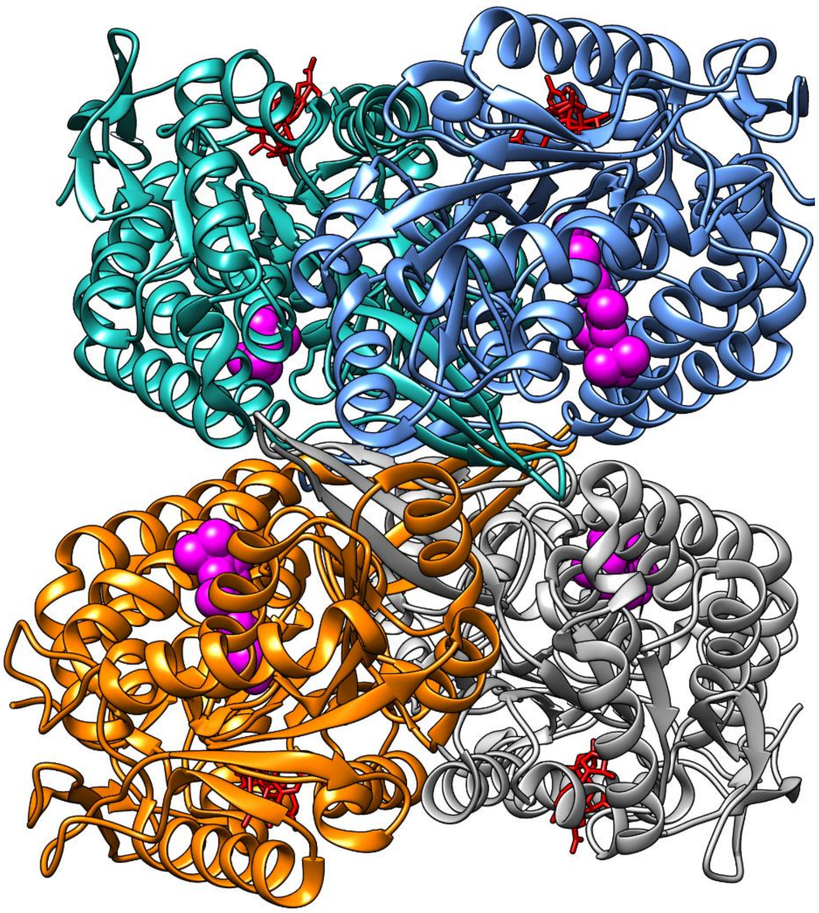

Figure S1. X-ray structure of tetrameric ALDH1A3. Chains A, B, C and D are colored in blue, cyan, gray and golden, respectively. Retinoic acid (RA) is shown as magenta spheres and NAD^+ as red sticks.

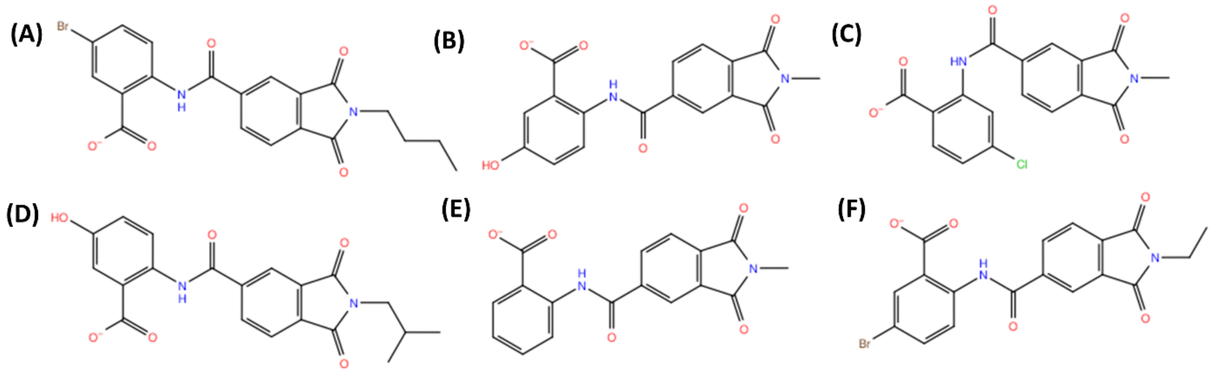

Figure S2. 2D chemical structures of virtual screening hits: **(A)** VS1. **(B)** VS2. **(C)** VS3. **(D)** VS4. **(E)** VS5. **(F)** VS6.

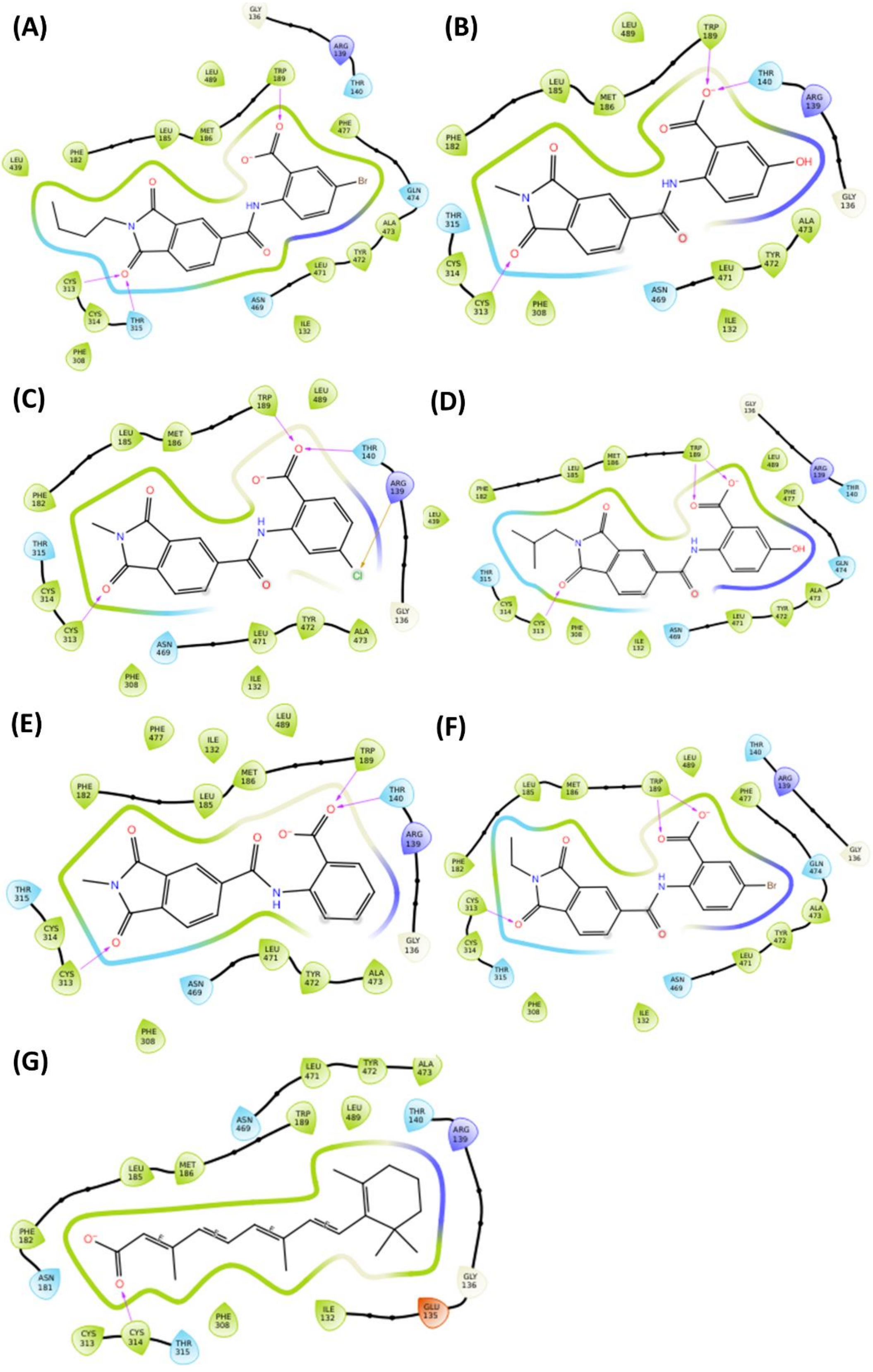

Figure S3. 2D protein – ligand interaction diagram of virtual screening hits. **(A)** VS1 **(B)** VS2 **(C)** VS3 **(D)** VS4 **(E)** VS5 **(F)** VS6 **(F)** RA in the ALDH1A3 binding site.

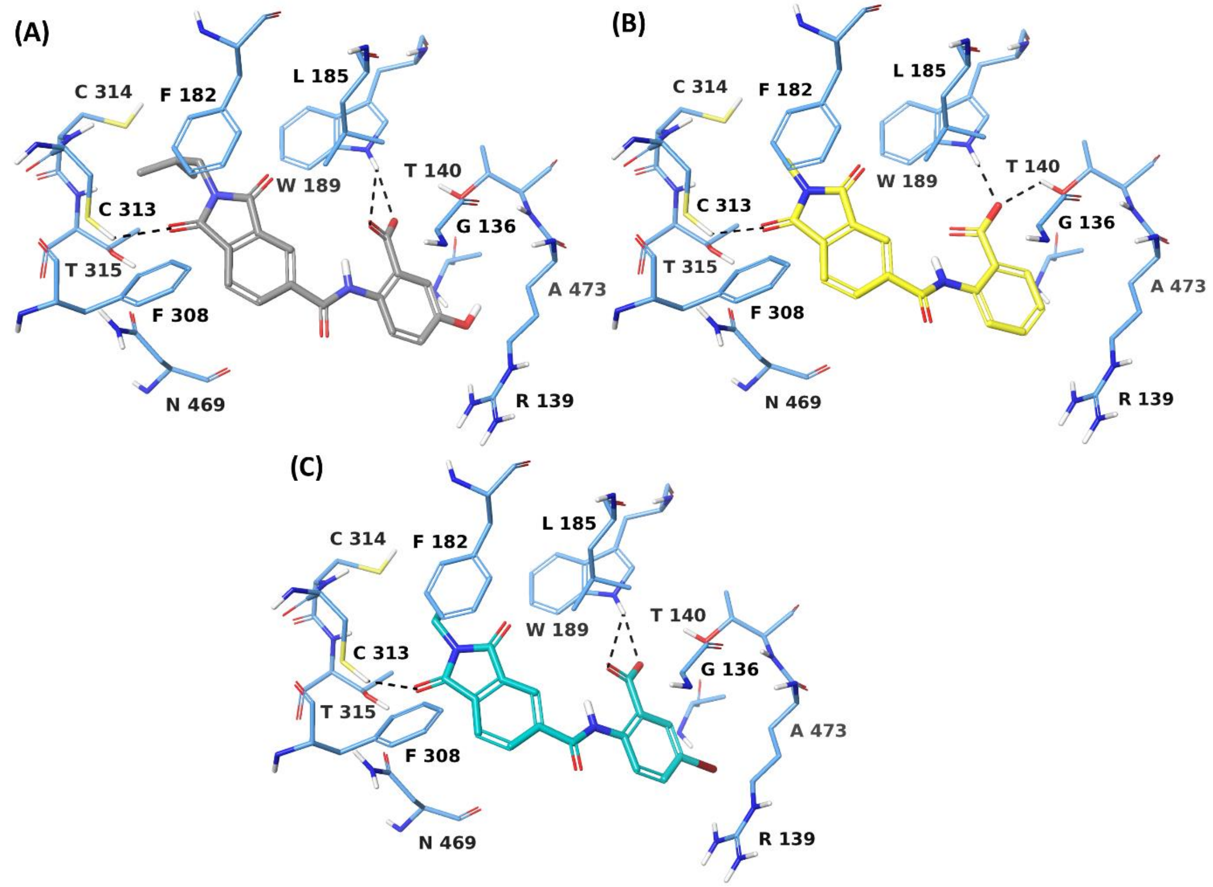

Figure S4. Docking pose of **(A)** VS4 **(B)** VS5 and **(C)** VS6 in the ALDH1A3 binding site.VS4, VS5 and VS6 are colored in grey, yellow and turquoise, respectively, while protein residues are in blue.

Table S1. Computed pharmacokinetic and physicochemical properties of the virtual screening hit compounds VS1 – VS6.

**Entry**	**VS1**	**VS2**	**VS3**	**VS4**	**VS5**	**VS6**
MW	445.26	340.29	358.73	382.37	324.29	417.21
LogPo/w	3.61	1.26	2.41	2.37	2.00	2.95
LogS	-5.54	-3.79	-4.66	-4.80	-3.86	-4.88
PSA	135.67	164.87	143.18	160.50	136.98	129.82
LogBB	-1.75	-2.45	-1.76	-2.54	-1.72	-1.48
CNS	-2	-2	-2	-2	-2	-2
LogKp	-4.03	-5.65	-4.83	-5.10	-4.35	-4.05
LogHERG	-3.90	-3.52	-3.58	-3.91	-3.66	-3.73
LogKhsa	0.13	-0.35	-0.14	-0.05	-0.26	-0.07
%HOA	73.88%	46.67%	61.99%	56.45%	62.16%	71.17%

MW: Molecular weight (<500) .

logPo/w: Octanol/water partition coefficient (<5).

logS: Aqueous solubility (-6.5 to 0.5 mol/L).

PSA: Polar surface area (7–200).

logBB: Predicted brain/blood partition coefficient (-3.0 to 1.2).

CNS: Predicted central nervous system activity (-2.0 to 2.0), -2 = completely inactive, -1 = very low activity, 0 = low activity, 1 = medium activity, 2 = completely active.

logKp: Predicted skin permeability (-8.0 to -1.0).

logHERG: Predicted IC_{50} value for blockage of HERG K+ channels (concern < -7).

logKhsa: Prediction of binding to human serum albumin (-1.5 to 1.5).

%HOA: Percent human oral absorption (>80% is high, <25% is poor).

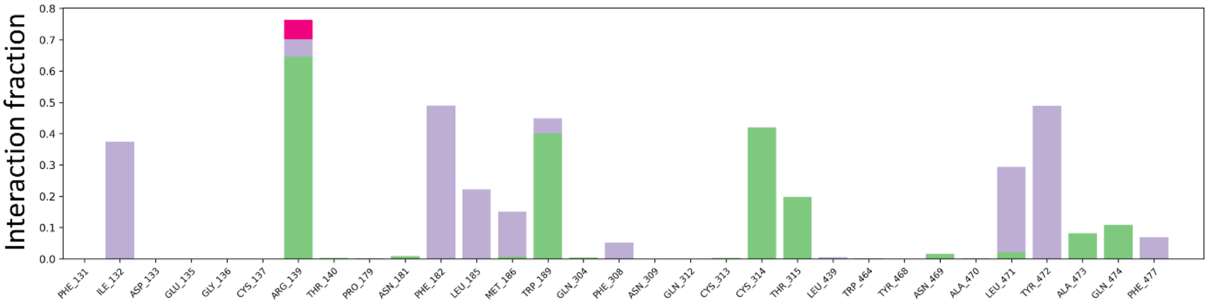

Figure S5. Protein-ligand interaction histogram from the MD simulations of VS1 in the ALDH1A3 active site. (H-bonds are shown in green, and lipophilic contacts in grey).

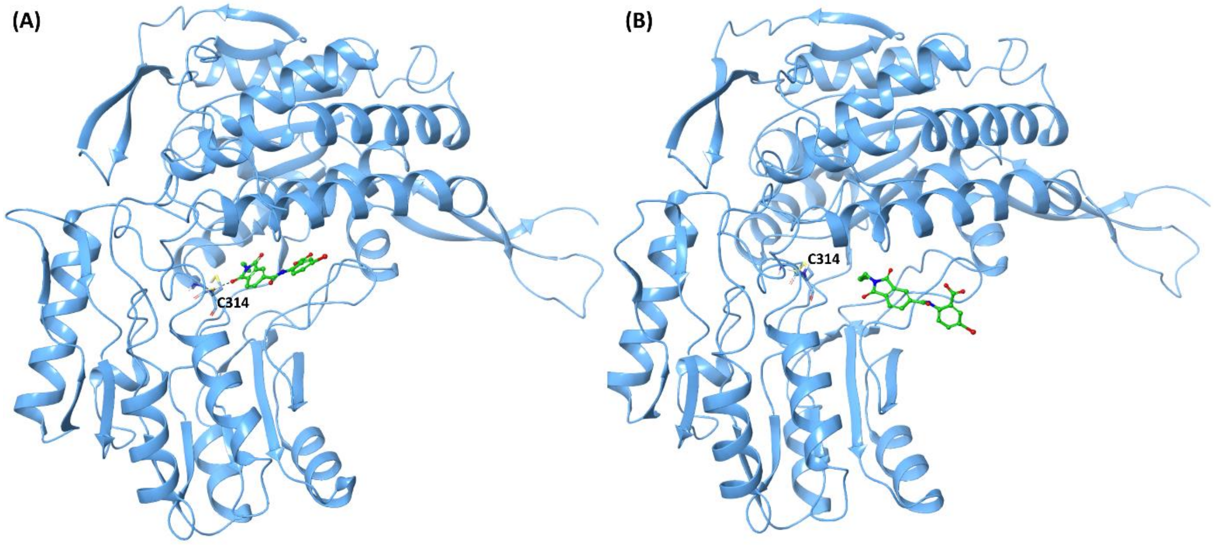

Figure S6. MD simulation trajectory snapshots of the ALDH1A3 -VS1 complex at **(A)** 58ns **(B)** 358ns. ALDH1A3 ribbons are shown in blue, while VS1 is shown in green.

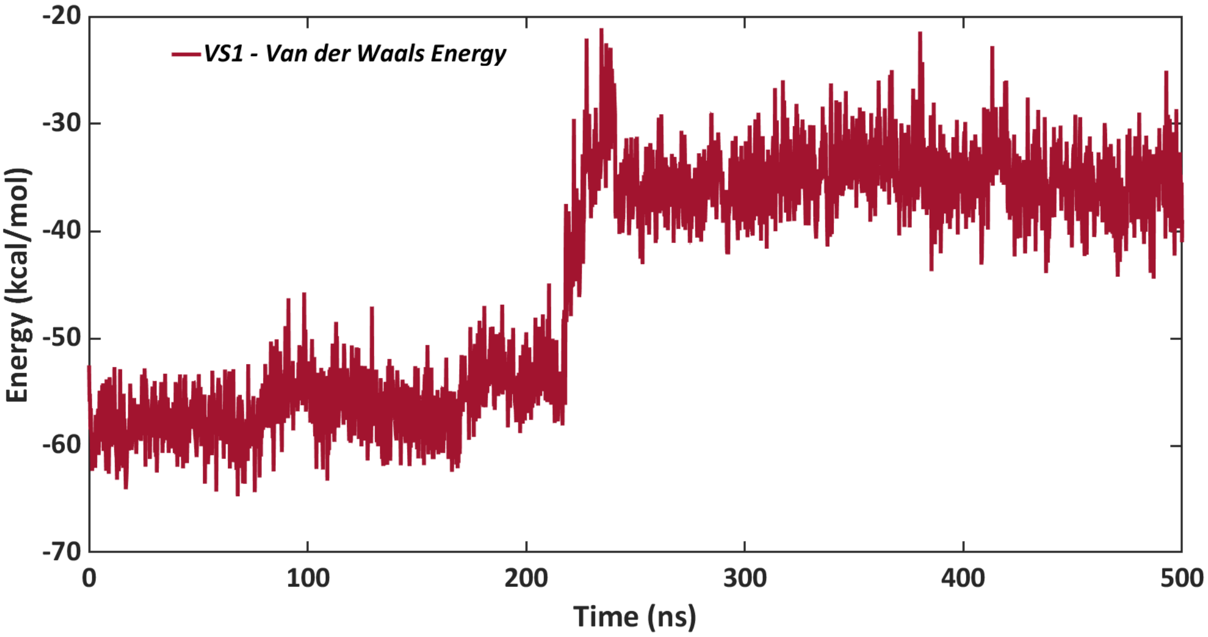

Figure S7. Van der Waals interaction energy contribution between VS1 and ALDH1A3 during the 500ns MD simulation.

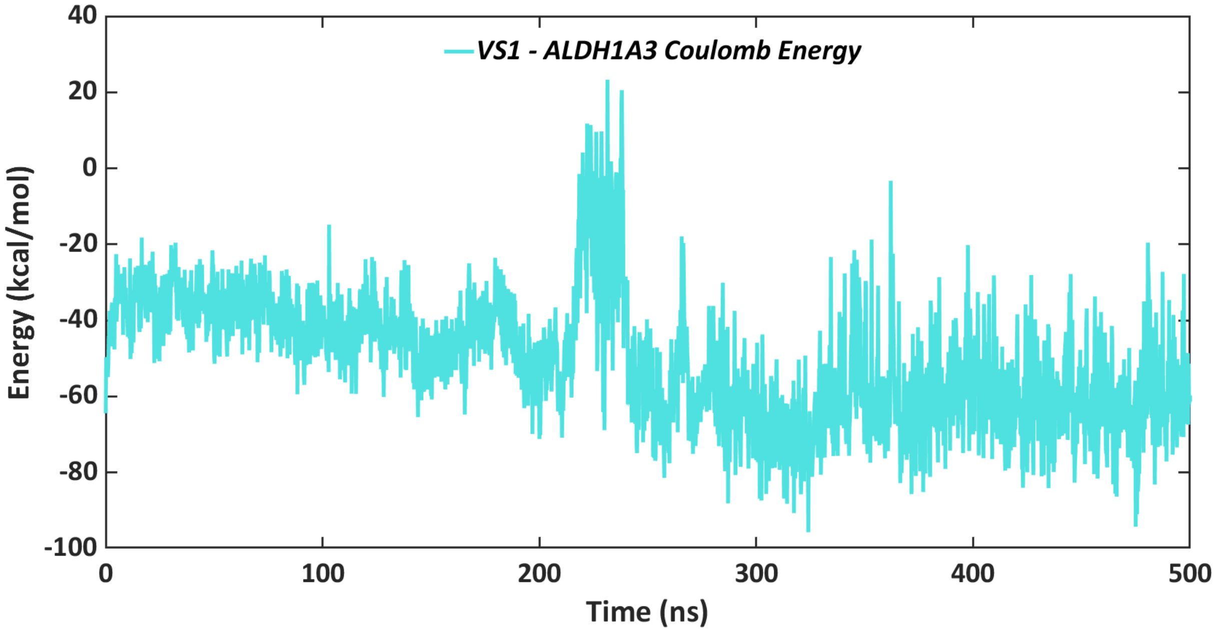

Figure S8. Coulomb interaction energy contribution between VS1 and ALDH1A3 during the 500ns MD simulation.

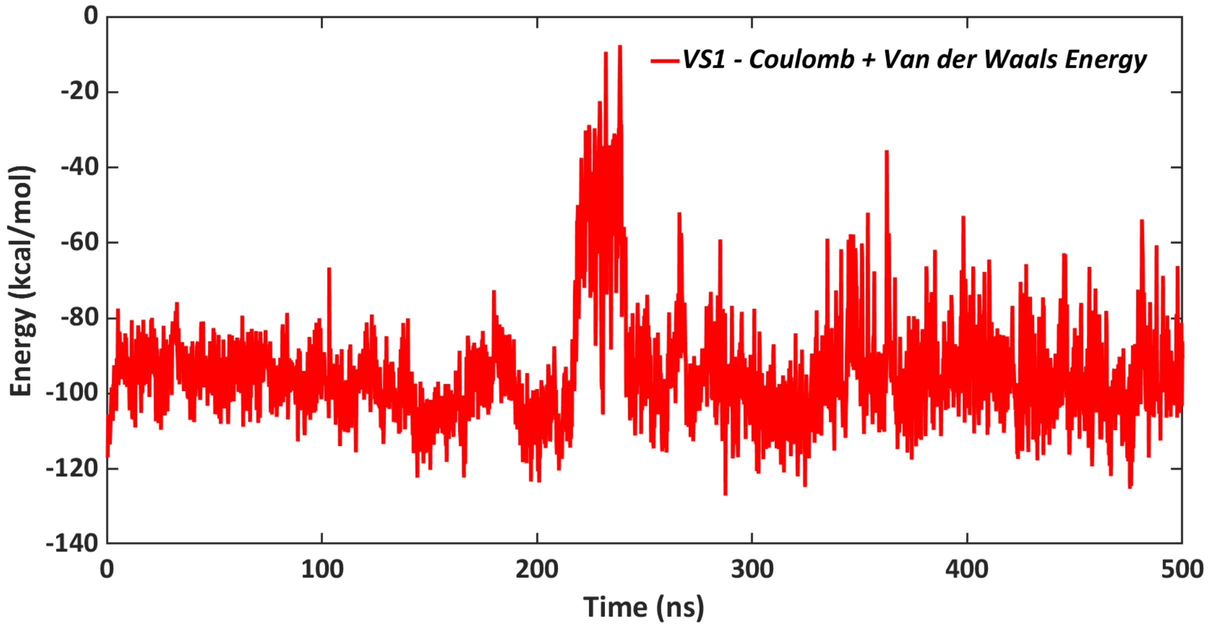

Figure S9. Total interaction (Van der Waals + Coulomb) energy contribution between VS1 and ALDH1A3 during the 500ns MD simulation.

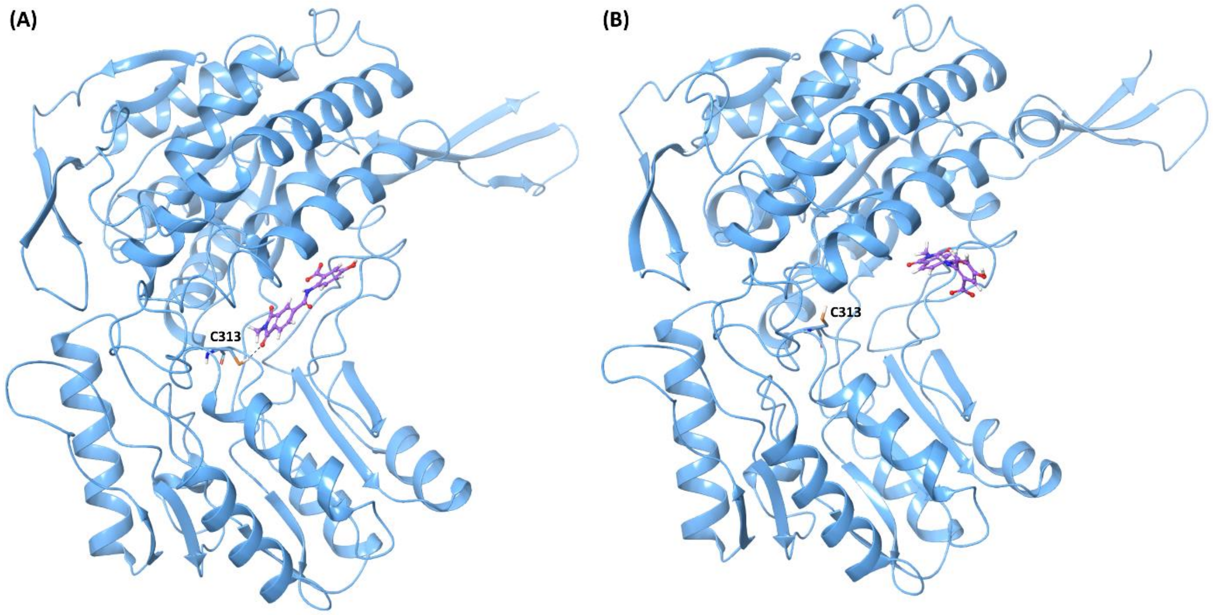

Figure S10. MD simulation trajectory snapshots of the ALDH1A3 -VS2 complex at **(A)** 0ns **(B)** 20ns. ALDH1A3 ribbons are shown in blue, while VS2 is shown in purple.

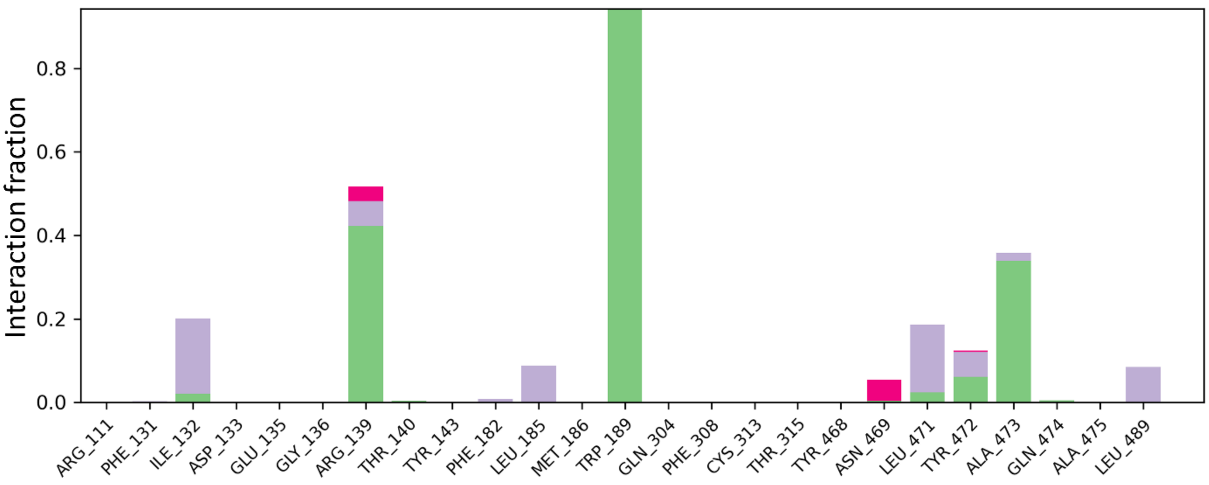

Figure S11. Protein-ligand interaction histogram from the MD simulation of VS2 in the ALDH1A3 active site. (H-bonds are shown in green, salt-bridge interactions in pink, and lipophilic contacts in grey).

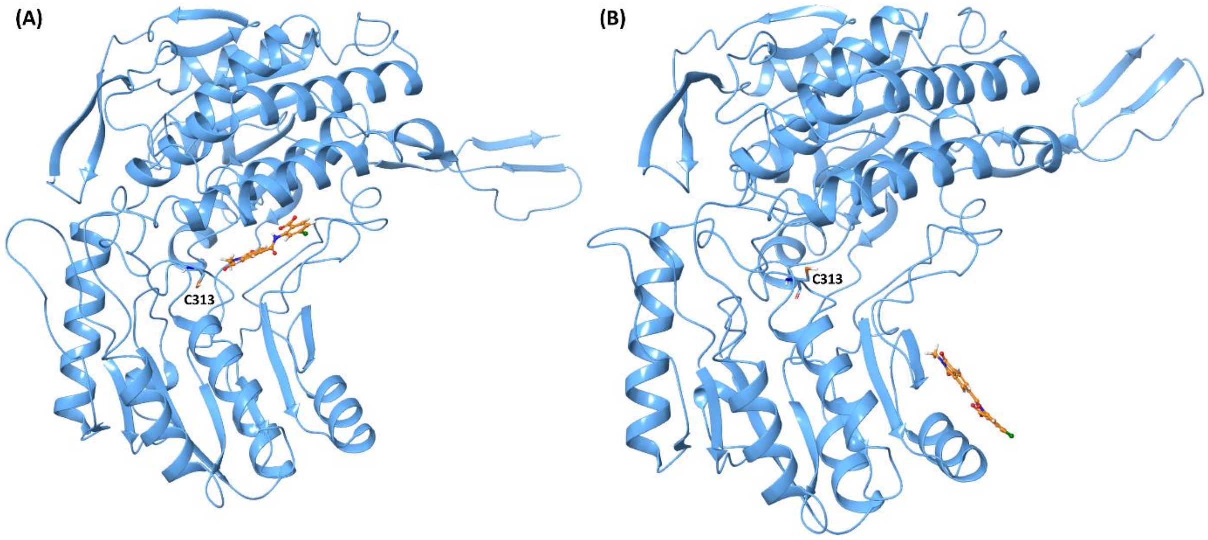

Figure S12. MD simulation trajectory snapshots of the ALDH1A3 -VS3 complex at **(A)** 0 ns **(B)** 20 ns. ALDH1A3 ribbons are shown in blue, while VS3 is shown in orange.

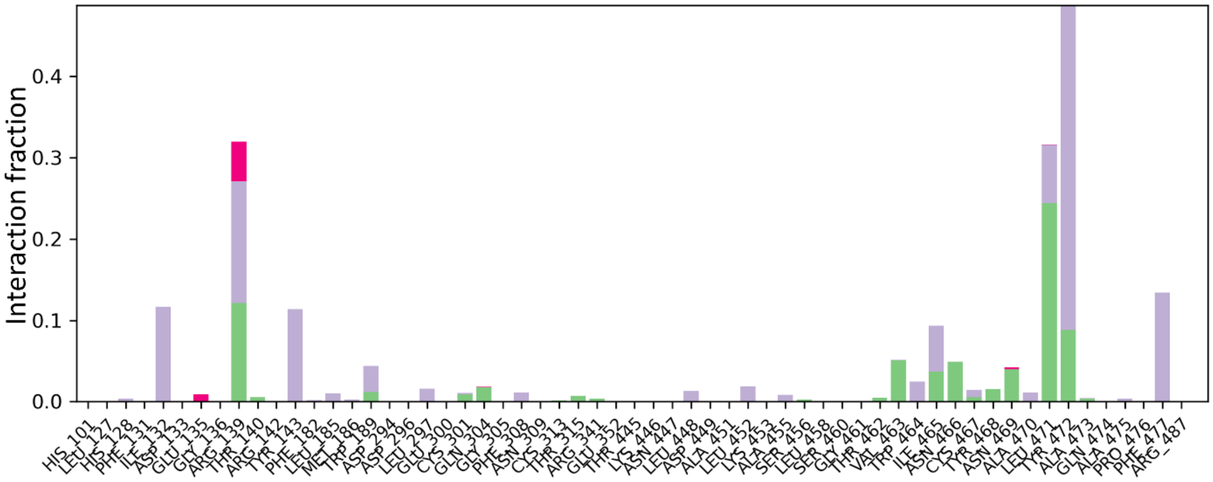

Figure S13. Protein-ligand interaction histogram from the MD simulations of VS3 in the ALDH1A3 active site. (H-bonds are shown in green, salt-bridge interactions in pink, and lipophilic contacts in grey).

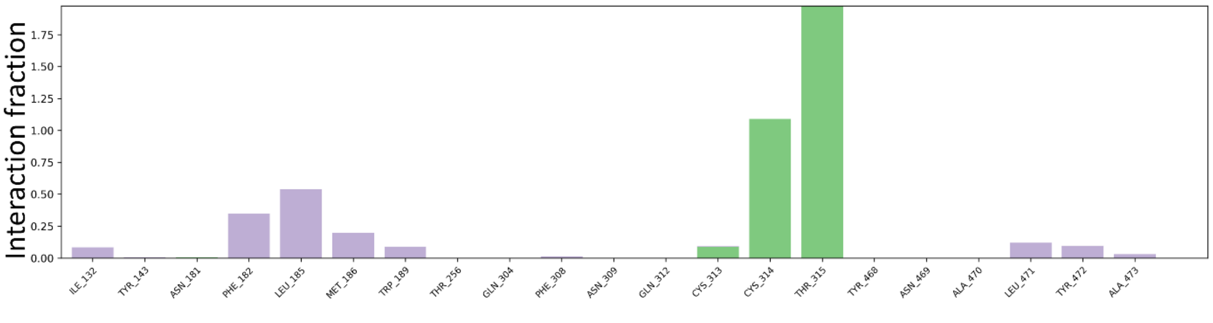

Figure S14. Protein-ligand interaction histogram from the MD simulations of Retinoic acid (RA) in the ALDH1A3 active site. (H-bonds are shown in green, and lipophilic contacts in grey).

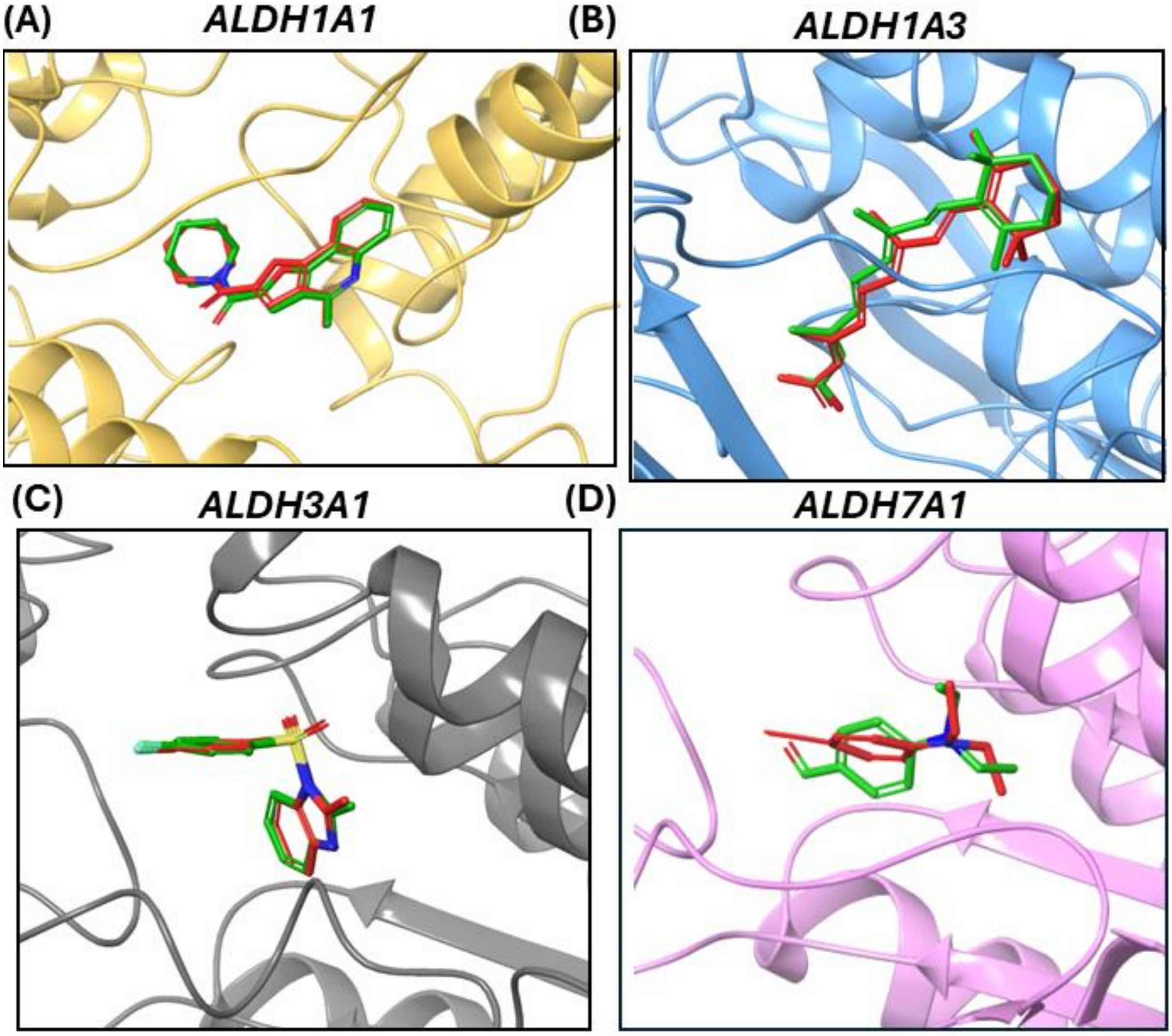

Figure S15. Glide SP redocking analysis of the co-crystallized complexes of **(A)** ALDH1A1 with CM38, **(B)** ALDH1A3 with RA, **(C)** ALDH3A1 with 1DD, and **(D)** ALDH7A1 with DEAB. The protein ribbons 1A1, 1A3, 3A1 and 7A1 are colored in yellow, blue, grey and pink, respectively, while the co-crystallized ligand and the redocked ligand are colored in green and red, respectively, in each case.

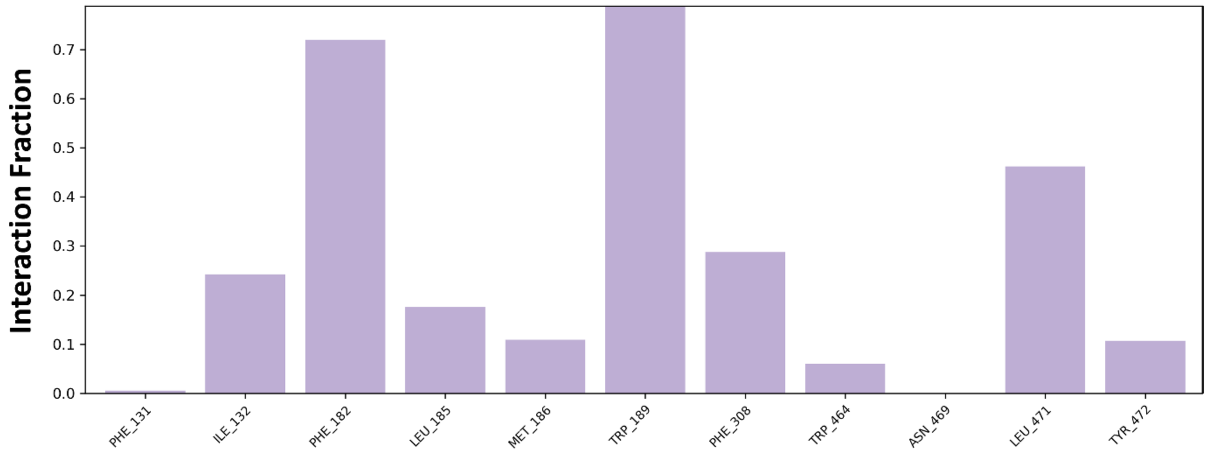

Figure S16. Protein-ligand interaction histogram from the MD simulations of MF13 in the ALDH1A3 binding site (Lipophilic contacts in are shown in grey).

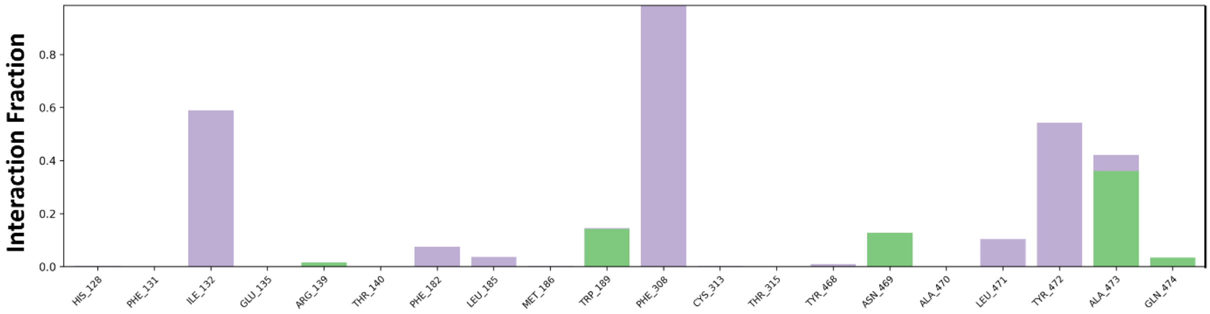

Figure S17. Protein-ligand interaction histogram from the MD simulations of MCI-INI-3 in the ALDH1A3 binding site (H-bonds are shown in green, and lipophilic contacts in grey).

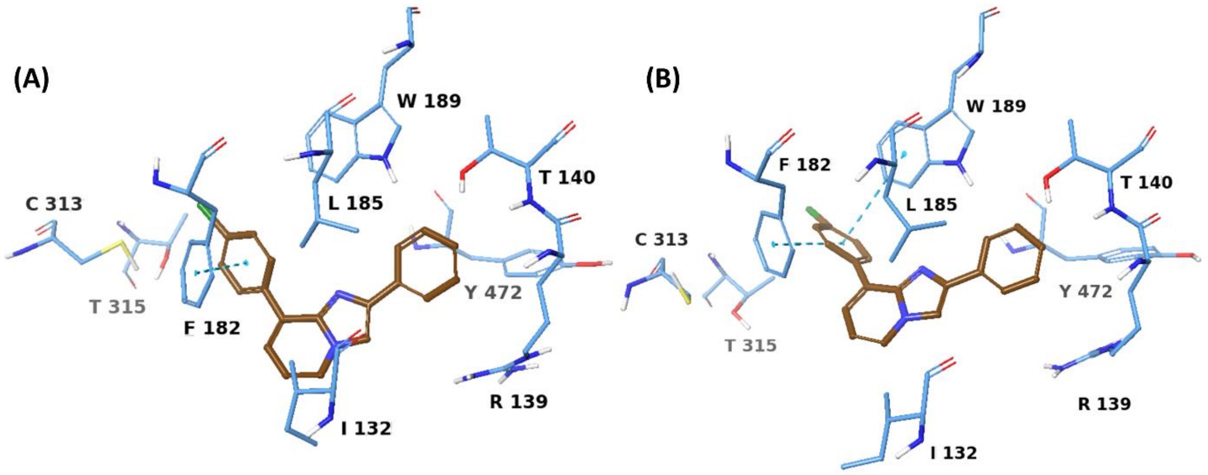

Figure S18. MD snapshots of MF-13 at **(A)** 164th ns, **(B)** 230th ns from the 500ns simulation trajectory. These snapshots show the conformation change taking place at the p-chlorophenyl ring of MF-13, facilitating lipophilic contacts with F182 and W189 at the ALDH1A3 binding site. MF-13 is colored in brown, while ALDH1A3 residues in blue.
